# PDZ Domains Across the Microbial World: Molecular Link to the Proteases, Stress Response, and Protein Synthesis

**DOI:** 10.1093/gbe/evz023

**Published:** 2019-01-29

**Authors:** Vijaykumar Yogesh Muley, Yusuf Akhter, Sanjeev Galande

**Affiliations:** 1 Instituto de Neurobiología, Universidad Nacional Autónoma de México, Querétaro, México; 2 Department of Biology, Indian Institute of Science Education and Research, Pune, India; 3 Department of Biotechnology, Babasaheb Bhimrao Ambedkar University, Lucknow, India

**Keywords:** PDZ, protease, evolution, translation, radical SAM, stress response

## Abstract

The PSD-95/Dlg-A/ZO-1 (PDZ) domain is highly expanded, diversified, and well distributed across metazoa where it assembles diverse signaling components by virtue of interactions with other proteins in a sequence-specific manner. In contrast, in the microbial world they are reported to be involved in protein quality control during stress response. The distribution, functions, and origins of PDZ domain-containing proteins in the prokaryotic organisms remain largely unexplored. We analyzed 7,852 PDZ domain-containing proteins in 1,474 microbial genomes in this context. PDZ domain-containing proteins from planctomycetes, myxobacteria, and other eubacteria occupying terrestrial and aquatic niches are found to be in multiple copies within their genomes. Over 93% of the 7,852 PDZ domain-containing proteins were classified into 12 families including six novel families based on additional structural and functional domains present in these proteins. The higher PDZ domain encoding capacity of the investigated organisms was observed to be associated with adaptation to the ecological niche where multicellular life might have originated and flourished. Predicted subcellular localization of PDZ domain-containing proteins and their genomic context argue in favor of crucial roles in translation and membrane remodeling during stress response. Based on rigorous sequence, structure, and phylogenetic analyses, we propose that the highly diverse PDZ domain of the uncharacterized Fe–S oxidoreductase superfamily, exclusively found in gladobacteria and several anaerobes and acetogens, might represent the most ancient form among all the existing PDZ domains.

## Introduction

Proteins displaying both signaling and protein–protein interaction domains are prevalent in eukaryotic signal transduction systems (Nourry et al. 2003). This domain architecture provides an elegant solution to regulate complex biological networks by sensing the incoming signals through effector domains, whereas the protein–protein interaction domains may amplify the signals (Manjunath et al. 2018). The PDZ domain is one of such protein–protein interaction domains. It was first identified in the context of signaling proteins, which are referred to as GLGF repeats proteins or DHR (discs large homology repeat) proteins (Cho et al. 1992; Ponting and Phillips 1995). The abbreviation PDZ was derived from the three metazoan proteins in which this domain is first reported: PSD-95, DLG, and ZO-1(Kennedy 1995).

Metazoan PDZ domains referred to as the canonical PDZ domains typically comprise 80–100 amino acid residues harboring a highly conserved fold (Kennedy 1995; Doyle et al. 1996). The typical secondary structures present in them are six β-strands with a short and a long α-helix, which may vary in different PDZ domain-containing proteins (Cabral et al. 1996; Doyle et al. 1996). However, the eubacterial PDZ domains fold similarly to the metazoan domains but with a distinct topology of secondary structural elements and are referred to as noncanonical (Harris and Lim 2001; Lee and Zheng 2010). Noncanonical PDZ domains consist of a circularly permuted structural fold, which means that they show variability in their primary amino acid sequence; however, their tertiary structure remains conserved. Nevertheless, all PDZ domains exhibit diversity in their functional roles and binding specificities (Sakarya et al. 2010; Belotti et al. 2013).

The origin, diversity, and functions of prokaryotic and fungal PDZ domains are largely unknown. The genome-wide analysis of nonmetazoan PDZ domains dates back to 1997, wherein these domains were shown to occur in bacteria (in abundance), plants, and fungi (Ponting 1997). However, its presence in fungi was considered doubtful due to low sequence similarity with known PDZ domains. Therefore, it was assumed either that the primordial PDZ domain arose prior to the divergence of bacteria or eukaryotes or that horizontal gene transfer led to the acquisition of these domains by bacteria (Ponting 1997). Even to date there are only a few reports which show the presence of these domains in fungi and archaea (Sakarya et al. 2010), whereas others suggest an absence (Lipinska et al. 1990; Harris and Lim 2001). It was hypothesized that a subset of eubacterial PDZ domain containing proteins might possess the precise canonical fold observed in metazoans, and the domain might have coevolved with multicellularity and organismal complexity (Harris and Lim 2001; Sakarya et al. 2007). However, the PDZ domain-containing protein-coding gene repertoire in prokaryotes and fungi was not explored in terms of their genomic organization, which may provide clues regarding their origin and evolution.

In the current study, we have identified, analyzed, and classified a complete repertoire of the PDZ domains in 1,474 prokaryotic and fungal genomes using cutting-edge remote homology detection techniques. We found that genomes of several eubacteria encode more than 15 PDZ domain-coding genes. These bacterial species exhibit features of complex traits and few also form multicellular communities. We have classified 93% of these proteins into 12 families based on conserved domain architecture, of which eight families are reported here for the first time, providing a glimpse of their ancestral history and functional clues. Furthermore, the genomic context analysis connects these genes to protein synthesis. This work bridges the ever-increasing gap between prokaryotic/fungal and metazoan PDZ domain studies and provides insights into the role of PDZ domains in the evolution of multicellularity.

## Materials and Methods

### Identification and Analysis of PDZ Domain-containing Proteins in Completely Sequenced Genomes

Protein sequence and annotation files (.faa and .ptt) obtained from the National Center for Biotechnology Information (NCBI) for completely sequenced prokaryotic and fungal species using ftp://ftp.ncbi.nih.gov/genomes/, last accessed November 2012 (Sayers et al. 2012). Out of a total of 2,057 genomes, we selected 1,474 representative genomes for which phenotypic information was available for further analyses. Genome size, taxonomy, and phenotype information for these species were obtained from NCBI microbial genome project files “lproks_0.txt” and “lproks_1.txt” which were available at ftp://ftp.ncbi.nih.gov/genomes/genomeprj/, last accessed November 2012 now retired. The obtained information is provided as a supplementary data set 2, supplementary file 3, Supplementary Material online. Hidden Markov models (HMMs) of PDZ domains were downloaded from the Pfam and Superfamily databases (Sonnhammer et al. 1997; Gough et al. 2001). The accession numbers for the Pfam domains are PF00595, PF13180, PF12812, and 50156 for the Superfamily database. These HMMs searched against all protein sequences of each selected genome using *hmmsearch* program from the HMMER package (Finn et al. 2011). The inclusion thresholds of *e*-value ≤0.01 and ≤0.03 were used to consider the significance of the output sequence and the obtained hit, respectively. The resulting sequences subjected to the *hmmscan* analysis to identify other domains using HMMs of complete Pfam and Superfamily databases. The *e*-value was set at 0.01 for HMM and 0.01 for the obtained hits. The output of *hmmscan* for both Pfam and Superfamily database search was analyzed separately using in-house Perl scripts and those available at http://supfam.org/SUPERFAMILY/downloads.html, last accessed November 2012, respectively, to extract the domain organization of each protein sequence. This search identified 7,852 protein sequences with at least one PDZ domain predicted using either Pfam or Superfamily HMM model. Subcellular localization of these proteins was predicted using Phobius web server (Käll et al. 2007). The data were processed using in-house Perl scripts and visualized over a pruned version of the NCBI taxonomy tree, which was created using interactive Tree Of Life (iTOL) webserver’s API tool by providing the NCBI taxonomy identifiers for investigated organisms (Letunic and Bork 2007).

### Classification of PDZ Domain-containing Proteins

The classification of PDZ domain-containing proteins is challenging, owing to their sequence and structural variations. On several instances, we were unable to find correspondence between the hits identified by the Superfamily and Pfam models due to the different classification strategies adopted by them. To overcome this problem, Pfam classification was used as a reference and was always cross-checked with Superfamily classification for consistency. First, we grouped proteins based on conserved Pfam domain architectures using in-house Perl scripts. The remaining sequences were manually checked and assigned to each group. Second, the *clustalo* program was used to construct a multiple sequence alignment (MSA) with default settings for each group, which were manually analyzed to exclude highly divergent sequences (Sievers et al. 2011). At multiple instances, a prototype motif of the specific family was considered for assigning proteins to their respective group (e.g., motifs highlighted in supplementary figs. 4 and 8, supplementary file 1, Supplementary Material online). This semiautomatic sequence analysis led to the classification of 7,318 out of 7,852 proteins into 12 families. We were unable to classify 7% proteins due to their presence in <20 species and highly variable domain combinations.

### Sequence, Structure, and Phylogenetic Analysis

PDZ domains inherently diverge at sequence and structure level. This hinders the phylogenetic signal in addition to its small length, leaving a few informative sites for phylogenetic reconstruction. Therefore, we selected PDZ domains only from the δ-proteobacteria group to reconstruct phylogeny. The selection was based on the presence of major classified proteins families in them. The *clustalo* program was used to align sequences using Superfamily HMM model, which is based on the alignment of PDZ domain structures. Positions that were conserved in more than 70% sequences were retained for analysis. We manually edited MSA to remove sequences that were highly divergent. Phylogenetic trees were reconstructed with Fitch–Margoliash and parsimony algorithms available through *fitch* and *protpars* programs in Phylip package, respectively (Felsenstein 1989). The statistical significance was accessed with 1,000 bootstraps. Protein distance matrix was constructed using the *protdist* program in Phylip package to feed in *fitch* program. A maximum likelihood tree was constructed using RAxML v. 8.1.24 (Stamatakis 2006), as implemented on the CIPRES web server (Miller et al. 2010), under the WAG (Whelan and Goldman) plus gamma model of evolution, and with the number of bootstraps automatically determined (MRE-based bootstrapping criterion). A total of 660 bootstrap replicates were conducted under the rapid bootstrapping algorithm, with 100 sampled to generate proportional support values. MrBayes analysis was performed for 1 million generations with WAG substitution model and a gamma distribution for four categories (Huelsenbeck and Ronquist 2001). The trees were sampled after every 1,000 generations and the first 25% were discarded as burn-in. The resulting trees were visualized in FigTree1.4 (available at http://tree.bio.ed.ac.uk/software/figtree/) with levels of support shown as posterior probabilities. The 3D structures were modeled using the Phyre2 web server (Kelley et al. 2015) and the PyMol software was used for analysis and visualization.

### Genomic Context Analysis

Protein annotation files were used to extract genomic coordinates of genes encoding the PDZ domain-containing proteins. We considered a gene as a neighbor if it was codirectional and placed within 50 nucleotide bases from a PDZ domain-coding gene on the genome (Muley and Ranjan 2012).

### Statistical Significance

The difference between two distributions of PDZ-containing proteins/domains was assessed using Wilcox rank sum test (Wilcoxon 1945). The null hypothesis was either no difference in two distributions or one distribution is greater than the other. The null hypothesis was rejected, and the difference considered significant if the Wilcox test *P*-value was lower than 0.05.

## Results

### PDZ Domains Are Ubiquitous Across the Microbial World

**Fig. 1.**
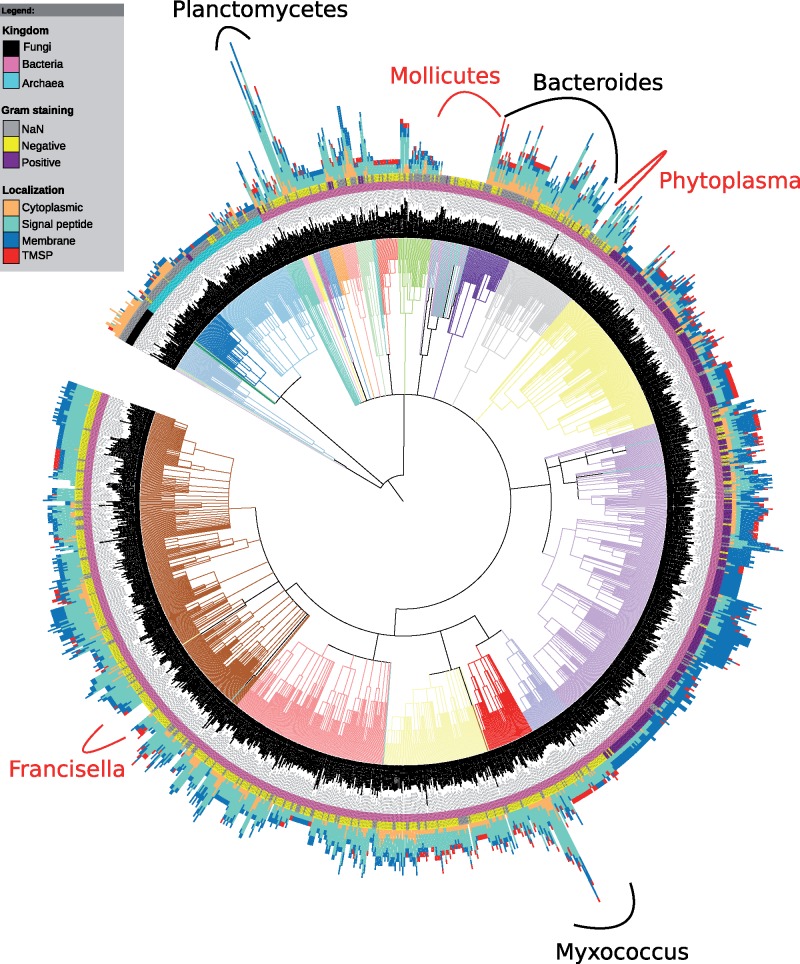
—The phylogenetic extent of PDZ domain-containing proteins across the microbial world. The distribution of PDZ domain-containing proteins is superimposed over the NCBI taxonomy tree using iTOL web server. The outermost layer depicts bar plots of a height proportional to the number of PDZ domain-containing proteins in a taxon. Each bar plot features four colors corresponding to the number of proteins in their four predicted subcellular locations. The innermost and middle color strips represent Gram nature and kingdom of taxa, respectively. Branches are colored to distinguish various classes/phyla. Lineages encoding a higher number and no PDZ domains are annotated in black and red colors, respectively. The distribution shows the ubiquitous occurrence of PDZ proteins across bacteria and scarcity in archaea and fungi. Higher number of membrane (TM) and secretory proteins (SP) are present in Gram-positive and -negative bacteria, respectively. Fungi encode mostly cytoplasmic PDZ proteins. TMSP stands for proteins predicted to have both transmembrane helix and signal peptide, and NaN for no information available.

**Fig. 2.**
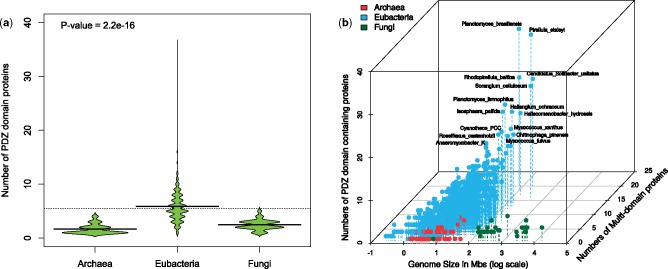
—A number of PDZ domain-containing genes within the individual genomes correlates with the size of genomes in eubacteria but not with archaea and fungi. (*a*) The bean plot demonstrates the distribution of proteins in the three domains. In the plot, the horizontal dotted line corresponds to the overall average number of proteins across all genomes under study. Bean-width corresponds to the proportion of genomes in it and bean-line shows the average number of proteins in the kingdom. Archaeal and fungal genomes encode <5 proteins on average, whereas many eubacteria encode substantially higher number of proteins. (*b*) The scatter plot illustrates the relationship between genome size, number of proteins, and multidomain proteins. Numbers of proteins and their multidomain architecture expanded with the increase in genome size in eubacteria. Proteins with repetitive PDZ domain alone were not counted as multidomain. The species with complex processes and/or the ability to form cell aggregates are annotated in the plot.

We identified 7,852 proteins in 1,419 of 1,474 (96%) analyzed genomes (fig. 1) with 9,975 significant hits to the PDZ domain HMMs obtained from the Pfam and Superfamily databases. Wherever more than one PDZ domain hits were observed, we considered them as tandem repeats. We observed that the PDZ domain-containing proteins were more abundant in the eubacterial species in comparison to the archaeal and fungal genomes (fig. 2). Eubacterial species encode PDZ domains in significantly larger numbers than archaea and fungi as revealed by the Wilcox test *P*-values 2.2e-16 and 2.769e-06, respectively (fig. 2*a*). Our results are partially consistent with previous findings; however, our analysis provided conclusive evidence toward the existence of the PDZ domains in archaea and fungi that was contested earlier (Ponting 1997; Harris and Lim 2001).

In the current work, PDZ domains were found absent in 4% of species (55 genomes), mainly belonging to cell-wall lacking mollicute and candidate phytoplasma species, of which many are adapted to a parasitic lifestyle (fig. 1). The loss of PDZ domains makes sense in these cell-wall lacking species as most of PDZ domain-containing proteins were predicted to have periplasmic or intramembrane subcellular localizations as shown in this work and also in several previous studies (Lipinska et al. 1990; Dartigalongue et al. 2001; Dong and Cutting 2004). Additionally, PDZ domains were also observed to be absent in *Buchnera aphidicola*. *Buchnera aphidicola* is reported to have shed all the genes encoding cell-surface components such as lipopolysaccharides and phospholipids; therefore, the absence of PDZ domain in the organism is congruent with the context (Shigenobu et al. 2000). The archaea, *Nanoarchaeum equitans*, also lacks any of the PDZ domains and it is reported to be completely dependent for lipids on its host *Ignicoccus hospitalis* (Jahn et al. 2008). These observations suggest that the lack of cell envelope components in these species is likely to have a correlation with the event of the absence of the PDZ domains in them. Therefore, we next studied the predicted subcellular localization of these PDZ domain-containing proteins. Consistent with our observations, 88.84% of the PDZ domain-containing proteins are predicted to have signal peptides (proteins targeted for secretion or to membrane compartments) and transmembrane helix or both, whereas the remaining 11.16% were cytoplasmic (supplementary fig. 1*a*, supplementary file 1, Supplementary Material online). These results further suggest that the eubacterial PDZ domain-containing proteins are mainly targeted to the membranes and membrane compartments (supplementary fig. 1*b*, supplementary file 1, Supplementary Material online), and could explain their absence in cell-wall lacking species. In contrast, archaeal and fungal proteins were mainly predicted to be cytoplasmic. Such localisation could be attributed to the diversified membrane architecture of archea and fungi as compared to the eubacteria (supplementary fig. 1b, supplementary file 1, Supplementary Material online) (Lombard et al. 2012).

Gram-negative and Gram-positive bacteria differ in their outer envelope architecture. Gram-negative bacteria possess a double membrane with periplasmic space between them compared with the single membrane in Gram-positive species (Cavalier-Smith 2006). If the presence of PDZ domains in bacteria is indeed associated with membrane components, then we expected that the number of PDZ domains would differ between these two classes of bacteria. Consistent with this, a significantly higher number of proteins with signal peptides occurred in Gram-negative species whereas transmembrane helices occurred more frequently in Gram-positive bacteria, with Wilcox test *P*-value 2.2e-16 (supplementary fig. 1*c*, supplementary file 1, Supplementary Material online). Additionally, Gram-positive species were also shown to possess significantly lower numbers of PDZ domain-containing proteins in comparison to the Gram-negative (Wilcox test *P*-value 1.925e-08). Collectively, our results indicate the wide occurrence of PDZ domains in prokaryotes and fungi. Their absence in certain species could have evolutionarily linkage to the loss of cell envelope components.

### The Evolution of Cellular Complexity and Multicellularity with the Expansion of PDZ Domains

**Fig. 3.**
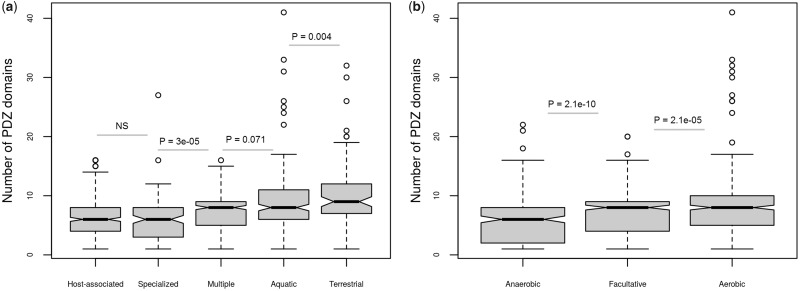
—Higher numbers of PDZ domains occur within a genome as the complexity level of organism increases. The boxplot depicts the distribution of PDZ domains in prokaryotes that are classified based on their (*a*) habitats and (*b*) oxygen requirement. Test of significance *P*-values using Wilcox test is shown above each of the two compared groups; null hypothesis was the number of PDZ domains in organisms belonging to right side group is higher than the left side. NS stands for nonsignificant *P*-value. Prokaryotes encoding a higher number of PDZ domains favor aquatic, terrestrial, and aerobic niche. In panel (*b*) “Multiple” stands for species with a wide host range and variety of habitats; “Host-associated” for species obligatorily associated with a host; and “Specialized” for those with specialized habitat, that is, environments such as marine thermal vents.

The numbers of PDZ domain-containing proteins and their multidomain architecture expanded with the increase in genome size across most of the organisms (fig. 2*b*). However, this trend is significant only for eubacterial genomes and not for archaea and fungi (supplementary fig. 2, supplementary file 1, Supplementary Material online). The abundance of PDZ domains notably differs across different habitats (fig. 3*b*). Terrestrial and aquatic bacteria encode higher numbers of PDZ domains in comparison to those inhabiting multiple changing environments; an obligatorily host-associated; and other habitats such as marine thermal vents (fig. 3*a*). Evidently, prokaryotes encoding higher number of PDZ domains are aerobic than anaerobic or facultative with Wilcox *P*-value 2.1e-05 (fig. 3*b*). Based on these results, it is tempting to speculate that the expansion of PDZ domain-coding genes might have some role in the adaptation of these PDZ domain-bearing organisms to the aerobic, aquatic and terrestrial lifestyles. Two other facts supporting this notion are that the metazoan life flourished in these habitats and PDZ is the second most abundant functional domain in animal proteins. PDZ being the second most abundant domain in metazoa also suggests that this domain might have coevolved with multicellularity and complexity of eukaryotes (Harris and Lim 2001; Kim et al. 2012). We observed more than ten PDZ domain-containing proteins in several species (figs. 1 and 2*b*), particularly in planctomycetes and myxobacteria—these species exhibit many unusual features normally absent in other eubacterial species. For instance, besides methylotrophic proteobacteria, only planctomycetes and myxobacterial members are able to synthesize C_30_ sterols such as lanosterol, which primarily occurs in eukaryotes (Pearson et al. 2003; Desmond and Gribaldo. 2009; Fuerst and Sagulenko 2011). Planctomycetes is a group of eubacteria possessing many features believed to exclusively occur only in eukaryotes such as the internal membranes, a primitive form of endocytosis, growth by budding, and lack of peptidoglycans (Pearson et al. 2003; Fuerst and Sagulenko 2011). Myxobacteria are known to form fruiting bodies, which behave in many aspects like a multicellular organism (Rokas 2008). Furthermore, proteins such as the Ser/Thr/Tyr protein kinases, which together comprise a major class of regulatory and signaling proteins in eukaryotes, were also reported in myxobacteria and planctomycetes (Perez et al. 2008). These results strengthen the hypothesis that the expansion of PDZ domains may have correlation with the evolution of cellular complexity and multicellularity.

### Classification and Cataloging of PDZ Domain-containing Proteins into 12 Families

In the literature, PDZ domain-containing proteins are classified as canonical and noncanonical based on the differences in their 3D structures obtained from metazoan and prokaryotic organisms, respectively. In contrast, the Superfamily database grouped PDZ domains into six families based on subtle differences in their protein structures, which are *PDZ domain*, the metazoan canonical form; *high-temperature requirement A-like serine protease* (*HtrA*), the noncanonical form abundant in prokaryotes*; tail-specific protease* (*Tsp*)*; EpsC C-terminal domain-like; MTH1368 C-terminal domain-like; and interleukin 16 domain-like* (supplementary fig. 3a, supplementary file 1) (Gough et al. 2001). Likewise, the Pfam database grouped domain sequences based on sequence similarity into three families: *PDZ*, the metazoan canonical form; *PDZ_2*, found in plants and eubacteria (noncanonical); and *PDZ_1*, found in few members of fungi and archaea (supplementary fig. 3b, supplementary file 1, Supplementary Material online) (Sonnhammer et al. 1997). As expected, the noncanonical HtrA superfamily (equivalent to the PDZ_2 domains in Pfam database) variant is the most widespread and abundant domain found across prokaryotes (supplementary fig. 3, supplementary file 1, Supplementary Material online). This observation is consistent with the previous findings (Ponting 1997). Harris and Lim (2001) have hypothesized the abundance of the canonical *PDZ domain* (equivalent to PDZ domain in the Pfam database) in bacteria, which might have contributed toward expansion and diversity in metazoan counterparts. Corroborating this hypothesis, we have identified several canonical PDZ domains in numerous bacteria (supplementary fig. 3, supplementary file 1, Supplementary Material online).

**Fig. 4.**
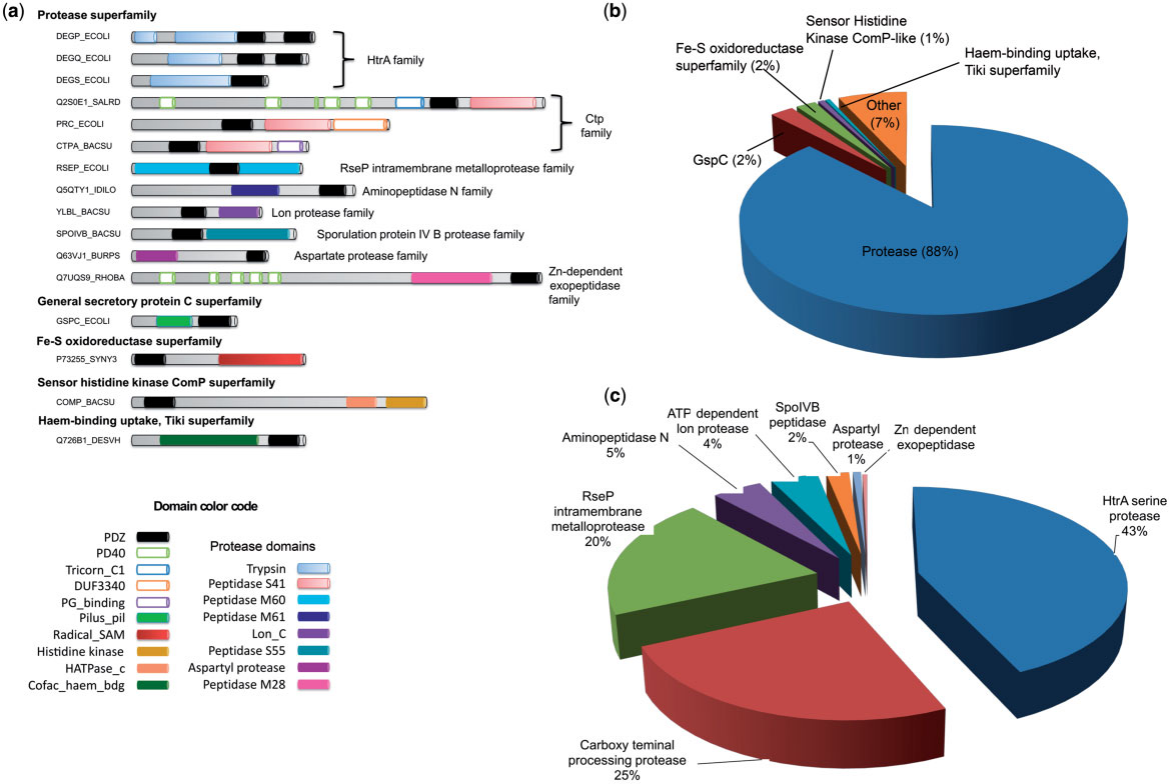
—Classification and cataloging of PDZ domain-containing proteins. (*a*) The panel depicts the most frequently observed domain architectures of the PDZ domain-containing proteins. Proteins are scaled approximately to the length of their primary sequence. Each domain architecture is a representative candidate of the superfamily/family/subfamily identified in this work. Protein identifiers are UniProt accession names. The Trypsin domain of the DEGP_ECOLI protein is interrupted by a Q-linker stretch. Protease domains are frequently combined with the PDZ domain. (*b*) The distribution of all PDZ domain-containing proteins into superfamilies identified in this study based on common domain architectures shown in (*a*). (*c*) The protease superfamily members are grouped into families based on protease type. Domain abbreviations: PD40, WD40-like beta propeller repeat domain; Tr_C1, tricorn protease C1 domain; Tr_PDZ, tricorn protease PDZ domain; PS41, peptidase family S41; DUF3340, C-terminal domain of tail-specific protease; PG_b, peptidoglycan-binding 1 domain; PM50, peptidase family M50; PM61, peptidase family M61; Lon_C, lon protease C-terminal proteolytic domain; PS55, peptidase family S55; Asp_prt, aspartyl protease domain; PM28, peptidase family M28; Pilus_P, type IV pilus biogenesis domain; Radical_SAM, radical SAM superfamily; HisKA_3, histidine kinase; HATPase_c, histidine kinase; DUF399, domain of unknown function.

PDZ domains exhibit an invariant 3D fold but are highly diverse at the sequence and secondary structure levels. It was challenging to analyze proteins at domain level due to different classification schemes adopted in literature, Superfamily, and Pfam databases. Therefore, we took into consideration the conservation of the PDZ domain and one of the other accompanying domains within the protein sequences and classified them in a semiautomated fashion (detailed in Materials and Methods). We successfully classified 93% (7,318 proteins) out of total 7,852 proteins into 12 families (fig. 4*a*, please see supplementary data set 1, supplementary file 2, Supplementary Material online, for individual proteins’ details). The domain architecture of the remaining 7% proteins was found to be conserved in <20 species and lacked any representative known proteins to confidently classify them into protein families. Therefore, we treated them as unclassified.

Figure 4*a* shows the domain architectures used for family-level classification of PDZ domain-containing proteins. A combination of PDZ and protease domains were observed most frequently in 88% of classified proteins. These 88% proteins were classified into eight different families based on the type of protease domain combined with PDZ. These families were referred to as HtrA, carboxy-terminal protease (Ctp), regulator of sigma-E protease (RseP), aminopeptidase N (APN), lon protease, sporulation protein IV B (SpoIVB), aspartate protease (AP), and Zn-dependent exopeptidases (ZEP) based on the annotation of protease domain present in them (fig. 4*c*). The PDZ domain in the remaining 12% proteins is combined with four nonprotease domains, and classified accordingly into four families, and are referred to as Fe–S oxidoreductase, general secretory protein C (GspC), Haem-binding uptake, and sensor histidine kinase ComP (ComP), based on the functions of the domains therein (fig. 4*b*). Previously characterized HtrA, Ctp, and RseP proteases were observed in high abundance compared with APN, lon protease, SpoIVB, AP, and ZEP family members. In spite of sequence and structural diversity, PDZ domains found to be preferentially combined with protease domain in 88% of classified proteins, suggesting its function is related to providing substrates for proteolysis. Consistently, the PDZ domains of HtrA, Ctp, and RseP were shown to recognize c-termini of unfolded proteins, which are processed by the accompanying protease domains in *Escherichia coli* (Anbudurai et al. 1994; Li et al. 2009; Clausen et al. 2011).

To note, eubacteria contain all these protein families; of which three also occur in archaea; and only HtrA family is present in fungi (Table 1). Multiple copies of HtrA and Ctp family proteins are found in eubacteria suggesting their expansion by gene duplication. We found that HtrA is the only family that exists in eubacteria, archaea, and fungi. The HtrA protein is a part of the elaborate high-temperature stress response system for protein quality control, which monitors protein homeostasis to prevent accumulation of unwanted and damaged proteins in the cell (Clausen et al. 2011). The occurrence of HtrA in organisms in all three domains of life suggests that their function is universally conserved. Novel findings related to few of the interesting families are discussed in detail in the subsequent sections.

**Table 1:** Taxonomic Distribution of PDZ Domain-Containing Protein Families Classified in This Work

Kingdom	Phylum	Htr	Ctp	RseP	**APN**^a^	**Lon**^a^	GspC	**FeSo**^a^	SpoIVB	**AP**^a^	ComP	**ChaN**^a^	**ZEP**^a^
Fungi		28/25	-	2/2^b^	—	—	—	—	—	—	—	—	—
	Ascomycota	28/25	—	—	—	—	—	—	—	—	—	—	—
	Basidiomycota	—	—	2/2^b^	—	—	—	—	—	—	—	—	—
	Microsporidia	—	—	—	—	—	—	—	—	—	—	—	—
Archaea		55/43	5/5^b^	109/108	13/13	—	—	—	—	—	—	—	—
	Euryarchaeota	27/18	2/2^b^	75/74	—	—	—	—	—	—	—	—	—
	Crenarchaeota	27/24	3/3^b^	33/33	13/13	—	—	—	—	—	—	—	—
	Nanoarchaeota	—	—	—	—	—	—	—	—	—	—	—	—
	Other Archaea	1/1	—	1/1	—	—	—	—	—	—	—	—	—
Eubacteria		2,902/1,271	1,716/1,041	1,262/1,242	294/246	292/290	182/170	156/148	139/136	49/48	48/41	37/37	29/25
	Acidobacteria	20/6	23/6	6/6	10/5	—	—	—	—	1/1	8/6	—	2/2
	Actinobacteria	302/138	31/24	117/116	1/1	113/113	—	5/5	—	—	—	—	—
	Alphaproteobacteria	470/146	165/128	146/145	29/19	—	11/11	—	—	2/2	—	1/1	—
	Aquificae	13/9	11/9	10/9	—	—	3/3	—	—	—	—	1/1	—
	Bacteroidetes/Chlorobi	81/63	265/63	63/63	33/22	—	—	—	—	22/22	1/1	—	7/7
	Betaproteobacteria	297/96	116/96	94/94	84/84	—	—	—	—	6/6	—	7/7	4/4
	Chlamydiae/Verrucomicrobia	21/20	24/20	20/20	—	—	—	—	—	—	—	—	—
	Chloroflexi	45/15	38/15	15/15	6/6	—	—	7/7	—	—	4/4	—	—
	Cyanobacteria	121/40	112/40	41/40	29/29	—	—	40/40	—	—	—	—	—
	Deinococcus–Thermus	47/13	30/13	13/13	—	—	—	6/6	—	—	—	—	—
	Deltaproteobacteria	120/43	77/43	52/43	10/7	—	23/21	9/9	—	5/5	—	17/17	4/4
	Epsilonproteobacteria	38/37	38/38	38/38	—	—	1/1	—	—	—	—	—	—
	Firmicutes	446/290	258/200	287/287	—	178/176	—	84/76	137/134	—	34/29	—	—
	Fusobacteria	4/2	5/5	4/4	—	—	—	—	—	—	—	—	—
	Gammaproteobacteria	663/282	416/270	282/275	85/66	1/1	137/127	—	—	10/10	1/1	8/8	3/3
	Other bacteria	65/23	39/25	26/26	1/1	—	1/1	5/5	2/2	1/1	—	3/3	—
	Planctomycetes	55/5	19/5	5/5	—	—	—	—	—	2/1	—	—	9/5
	Spirochetes	82/31	37/29	31/31	6/6	—	6/6	—	—	—	—	—	—
	Thermotogae	12/12	12/12	12/12	—	—	—	—	—	—	—	—	—

Note.—A protein family is shown by its total number of members/total number of organisms in particular taxonomic group. Protein families are abbreviated as: Htr, high-temperature requirement proteases; Ctp, C-terminal processing proteases; RseP, RseP intramembrane metalloproteasese; APN, aminopeptidase protein N; Lon, ATP-dependent lon proteases; SpoIVB, sporulation factor IV B proteases; AP, aspartate proteases; ZEP, Zn-dependent exopeptidases; GspC, general secretion pathway protein C; FeSO, Fe–S oxidoreductases; ComP, competence protein family; ChaN, EreA-Chan-like family.

a Uncharacterized families.

b Rare incidences of family proteins in respective group/kingdom which were considered as horizontally transferred events from eubacteria.

#### Recent Divergence of the PDZ Domain of Ctp Family: A Root of Metazoan Canonical PDZ Domains?

Of all, Ctp family is particularly interesting due to the presence of a canonical PDZ domain, which could be ancestral to the metazoan PDZ domains. At the functional level, the PDZ domain of a Ctp recognizes four hydrophobic residues at the C-terminus of the FtsI precursor, which is subsequently cleaved by the accompanying Peptidase_S41 domain and produces mature FtsI in *E. coli* (Hara et al. 1991) (fig. 4*a*). The PDZ and Peptidase_S41 domain combination is observed in 1,721 proteins. Along with these two domains, these proteins also possess domain of unknown function 3340 (DUF3340), peptidoglycan-binding domain, tricorn and tricorn_C1 domains in a lineage-specific manner (fig. 4*a*). This suggests that the Ctp family is amenable to evolutionary changes, and associated domains provided new functional aspects to it in a lineage-specific manner.

The canonical PDZ domain was detected based on Pfam as well as Superfamily HMM in 309 (out of 316) DUF3340 domain-containing family proteins in 298 species (supplementary table 1, supplementary file 1, Supplementary Material online). Interestingly, the DUF3340 domain shows sequence similarity with the mammalian interphotoreceptor retinoid-binding protein (IRBP) (Keiler and Sauer 1995) and might be ancestral to it (Ponting 1997). We searched the DUF3340 region of *E.coli* Ctp (Prc) in the UniProt database and found similar sequences in five archaeal and many metazoan species including the early branch point’s demosponge, sea anemone, and in human. However, we were unable to detect similar sequences in fungi and ecdysozoa members—worm and fly. This suggests the possible horizontal transfer of eubacterial PDZ domains to metazoa along with DUF3340 (IRBP). Phylogenetic analysis discussed in subsequent sections confirms the recent divergence of the PDZ domains of Ctp family.

#### Early Divergence of the PDZ Domain of the Uncharacterized Fe–S Oxidoreductase Superfamily: The Base of PDZ Domain Evolution

The PDZ domains in Fe–S oxidoreductase and GspC superfamily members were predicted with the Superfamily HMM but not with Pfam. This indicates that the sequence of the PDZ domain in these proteins diverged highly but the structure remained conserved. This could be the result of mutations accumulated over time, and hence one of the PDZ domains from these families could be the earliest diverged member from its ancestor. The GspC superfamily members are composed of PDZ and Pilus_Pil domains. GspC is a part of the type II secretion machinery (also referred to as the general secretory system) and is well characterized in the *E.coli* (Francetic et al. 2000). As the general secretory system is not specific toward a certain substrate, it naturally imparts fewer constraints on the sequence conservation of the proteins involved. GspC family proteins are mostly present in proteobacteria, which is not accounted among old bacterial phyla so far. On the other hand, radical *S*-adenosyl-l-methionine (SAM) domain is observed in 156 uncharacterized proteins in 148 eubacterial species. These proteins are referred to as the Fe–S oxidoreductases due to the highly conserved iron–sulfur binding motif and radical SAM domain. The PDZ domain occurs at the N-terminus of radical SAM domain (with a CxxxCxxC conserved motif) in these proteins (supplementary fig. 4, supplementary file 1, Supplementary Material online).

**Fig. 5.**
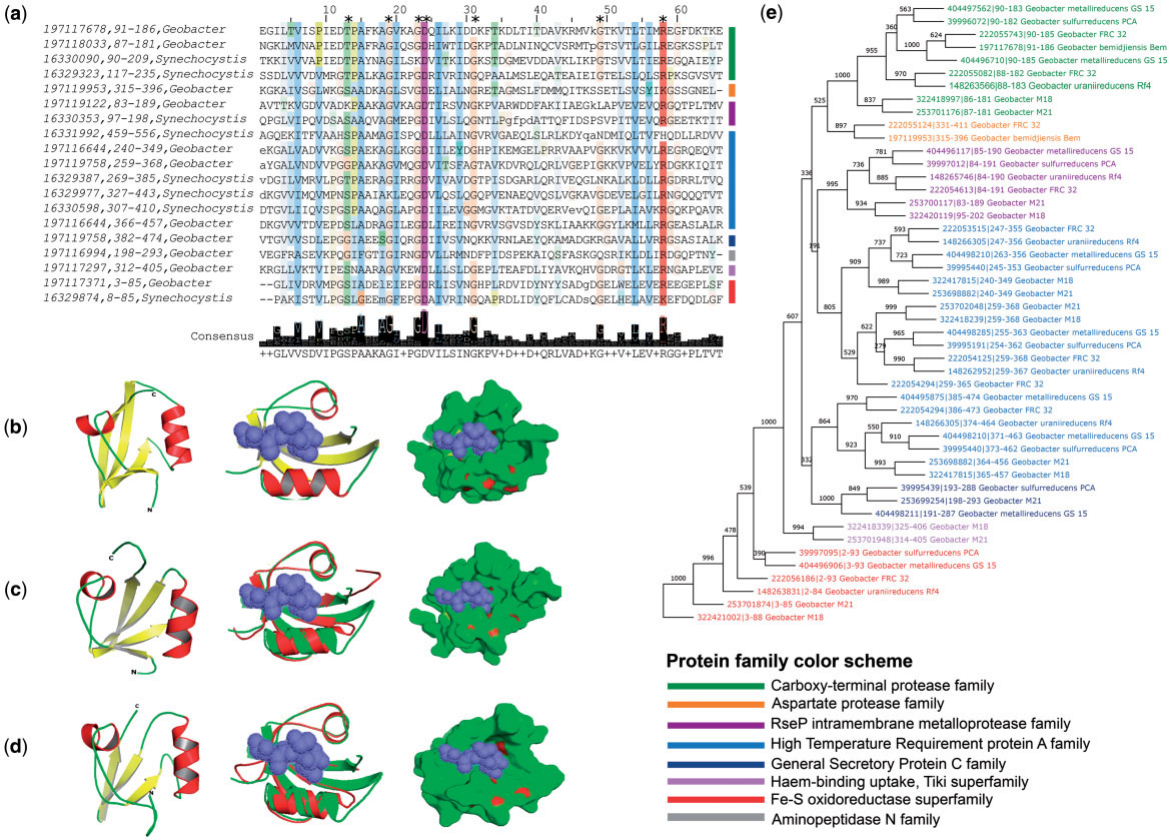
—Structure, function, and evolution of uncharacterized Fe–S oxidoreductase superfamily. (*a*) Multiple sequence alignment. (*b*) The structure of the PDZ domain of HtrA2 protein from *Mycobacteria* is shown without and with the tetra-peptide (Gly–Ala–Thr–Val) ligand. The predicted structure of PDZ domain from *Geobacter* (*c*) and *Synechocystis* (*d*). Their overlap with HtrA2 is shown along with its ligand. HtrA2 is shown in green color, whereas predicted structure in red. Phylogenetic tree shown in panel (*e*) is constructed using the Fitch neighbor-joining algorithm. Positions with asterisk marks on top in MSA are also conserved in metazoan PDZ domains. The predicted structure shows high overlap in the ligand-binding cavity. The phylogenetic tree shows ancestry of Fe–S oxidoreductases to other PDZ domains, whereas a recent divergence of the Ctp family domain.

The radical SAM domain is considered to be among the oldest domains. The highly conserved CxxxCxxC motif in them is likely to form an iron–sulfur (Fe–S) cluster to cleave SAM reductively and produce a radical (usually a 5´-deoxyadenosyl 5´-radical) (Frey et al. 2008). The radical intermediate allows a wide variety of unusual chemical transformations. These proteins are conserved in all cyanobacterial genomes investigated in this study, and half of the chlorobacteria and halobacteria (Table 1). These three phyla constitute the oldest bacterial clade—gladobacteria and a probable root of life placed in chlorobacteria (Cavalier-Smith 2006). These proteins are also present in firmicutes, δ-proteobacteria; and few actinobacterial species, which predominantly use anaerobic mode of respiration (Table 1). The presence of this family’s proteins in photosynthetic and anaerobic eubacteria suggests their functional role in the early earth environment (Lane and Martin 2012) and is consistent with their high sequence divergence due to the lengthy evolutionary time span. Furthermore, it occurs in several acetogens that generate acetate as a product of their anaerobic respiration and they have been proposed as the last universal common ancestor (Weiss et al. 2016). MSA analysis confirms the authenticity of these PDZ domains following structural analysis (fig. 5*a*). The protein sequence of slr2030 and GSU1997 hypothetical proteins of Fe–S oxidoreductase superfamily from *Synechocystis* PCC 6803 and *Geobacter sulfurreducens* PCA was used to predict the 3D structure using the Phyre2 protein modeling web server. The 61% N-terminus amino acids of both sequences modeled with more than 90% confidence level, which includes PDZ and radical SAM domain. The predicted structures were compared with the known ligand-bound PDZ domain structure of the HtrA2 protein isolated from *Mycobacterium tuberculosis* (MohamedMohaideen et al. 2008) (fig. 5*b*). The predicted domain structure is composed of two helices and three to four β-strands as opposed to around 5–6 β-strands often occurring in known structures (fig. 5*c* and *d*), indicating the insertion of additional β-strands in the PDZ domains of other families later during the evolution. Strikingly, even with only three β-strands, the peptide-binding cavity in the domain is well maintained suggesting its functional equivalence to other PDZ domains (fig. 5*c* and *d*). The overlap between predicted PDZ structures of slr2030 and GSU1997 proteins with the HtrA2 (PDB id 2z9i) is very high with root-mean-square deviation (RMSD) of 0.35 and 0.61, respectively. Next, we performed phylogenetic analysis to infer the divergence and origin of the domain. The PDZ domains of Fe–S oxidoreductases were consistently placed at the root of the tree by four different phylogeny reconstruction methods, whereas the Ctp PDZ domain was placed at the tip suggesting its recent divergence (fig. 5*e* and supplementary figs. 5–7, supplementary file 1, Supplementary Material online). On the other hand, GspC family domains may have diverged later from HtrA family domains.

Collectively, phylogenetic analyses strongly indicate that the PDZ domain of radical SAM proteins is presumably an ancestral form that might have given rise to other PDZ domains and recent divergence of the Ctp family domain. Phyletic distribution also supports the occurrence of the PDZ domains of radical SAM proteins in the last universal common ancestor.

### PDZ Domains Encoding Gene Cluster Analysis: Connection with Protein Synthesis and Membrane Remodeling

**Fig. 6.**
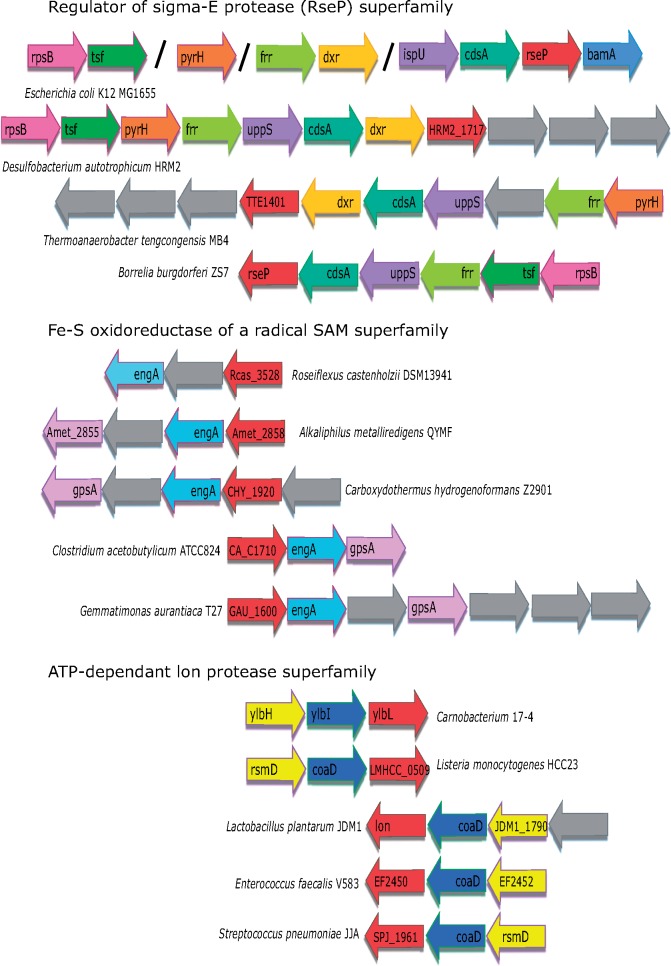
—Genomic context reveals colocalization of PDZ encoding genes with translation-associated genes. (*a*) *RseP* family members are frequently associated with *cdsA, uppS (ispU), dxr, frr, rpsB, and bamA* genes. Intergenic distance >50 nucleotides is indicated by a slash (/). (*b*) Fe–S oxidoreductases of radical SAM family members occur in the vicinity of *engA (der) and gpsA* genes. The gene between *engA and der* is conserved in many species, but no function is defined for it. (*c*) ATP-dependent lon protease family members are well conserved with *coaD and rsmD* gene. The *rsmD* gene is not well annotated in many genomes. Proteins encoded by *dxr, rpsB, frr, der, and rsmD* are associated with translation-related functions. The genes are included in this analysis if they are placed within a distance of 50 nucleotides from the PDZ-coding gene with the exception of *Escherichia coli* where it was 300 bases (*a*). Locus identifiers were used when gene names were not available. The red color is used to represent PDZ domain-containing proteins in each panel. Homologous genes are represented in the same color. Gray-colored genes are not conserved in the neighborhood. Arrows pointing toward the right represent the plus strand and toward the left represent the minus strand of the genome.

Conserved chromosomal colocalization of genes reflects their coregulation and often their products participate in the same or related functions (Lathe et al. 2000; Muley and Ranjan 2013). The genomic neighborhood of genes encoding the PDZ domain-containing RseP, ATP-dependent lon proteases (in *Bacillus subtilis* known as YlbL), and Fe–S oxidoreductase family is conserved in phylogenetically diverse species (fig. 6). RseP is an inner membrane metalloprotease that induces stress response via σ^24^ (RpoE) factor upon its activation by the HtrA family protein DegS in response to damaged outer membrane proteins in *E.coli* (Dartigalongue et al. 2001; Li et al. 2009). Characteristic features of this family’s proteins include two highly conserved motifs, HExGH and NxxPxxxLDG at their N- and C-termini, which are similar to the potential zinc-binding site found in a variety of metalloproteases (Brown et al. 2000) and to the motif found in the human S2P protease, respectively (supplementary fig. 8, supplementary file 1, Supplementary Material online) (Dartigalongue et al. 2001). The proximal genes of *RseP* mostly encode proteins responsible for outer membrane biogenesis and translation. These include *bamA* (OMP biogenesis) (Rhodius et al. 2006), *cdsA* (lipid biosynthetic pathway), *uppS* (or *ispU*, essential for carrier lipid formation in bacterial cell wall synthesis) (Kato et al. 1999), *dxr* (upstream of *uppS* catalyzed pathway), *rpsB* (part of the 30S ribosomal subunit), and *frr* (ribosome recycling factor upon translation termination) (Hirokawa et al. 2004) (fig. 6*a*). The presence of the *frr* gene is especially interesting because some of its mutants rapidly decrease protein synthesis followed by inhibition of RNA synthesis at 42 °C (Hirokawa et al. 2004). Mutations in the *rseP* characteristic motifs also exhibited lethality at temperatures above 41 °C (Dartigalongue et al. 2001). The upstream regions of both *rseP* and *frr* genes harbor a σ^24^ promoter in *E.coli*, suggesting their coregulation (supplementary fig. 9, supplementary file 1, Supplementary Material online). Therefore, we propose a possible link between translation and RseP functions in the high-temperature stress response. σ^24^ is positively regulated by the starvation alarmone ppGpp (guanosine 3′-diphosphate 5′-diphosphate) during entry into the stationary phase, suggesting that σ^24^ can respond to internal signals as well as stress signals originating in the cell envelope (Costanzo and Ades 2006). We propose that the internal signal through ppGpp might be responsible for the σ^24^ activation to maintain misfolded and damaged proteins during the stationary phase and for inhibition of *tff-rpseB-tsf* gene expression to cease translation due to nutrient limitations, because ppGpp leads to inhibition of expression of this transcription unit under amino acid starvation (Aseev et al. 2014). These observations associate the RseP family proteins directly with translation and membrane biogenesis.

The genomic context of the Fe–S oxidoreductase family members contains *gpsA* (encodes a glycerol-3-phosphate dehydrogenase enzyme) and *engA* (fig. 6*b*). The *engA* gene encodes a GTPase also called Der, which is required for ribosome assembly and stability. It cotranscribes with the outer membrane protein, BamB coding gene from a σ^24^ promoter in *E.coli* (Rhodius et al. 2006). The product of *bamB* is part of the large multiprotein BAM complex responsible for outer membrane biogenesis, including *bamA* sharing genomic context with *rseP* (fig. 6*a*). Interestingly, the radical SAM domain is predicted to possess a 3D fold similar to the SAM methyltransferase involved in translation. The genomic neighborhood of this family along with Der further strengthens our hypothesis that the Fe–S oxidoreductase family is likely to be involved in translation-related functions.

The PDZ domain combined with ATP-dependent lon protease in 292 uncharacterized proteins of actinobacteria and firmicutes (Table 1). A prototype example of such domain organization is the *B.subtilis* membrane protein YlbL, which exhibits a structural fold similar to the ribosomal protein S5. The genes encoding these proteins share their genomic neighborhood with *rsmD* (fig. 6*c*). The rsmD product plays a critical role in the methylation of G966 of 16S rRNA (Weitzmann et al. 1991), which modulates the early stages of translation initiation (Burakovsky et al. 2012). Substitutions at G966 generally result in virtually no effects on cell growth (Jemiolo et al. 1991) but does result in effects on stress response (Burakovsky et al. 2012). It suggested that RsmD acts in a complex way to shape the bacterial proteome under stress conditions. The null mutants of the rsmD gene remain in lag phase for prolonged periods under stress (Christensen et al. 2001; Burakovsky et al. 2012). Interestingly, this complements the inhibitory effect of lon proteases on translation, particularly during nutritional stress (Christensen et al. 2001).

## Discussion

The present study proposes for the first instance, the comprehensive classification and cataloguing of 93% of the PDZ proteins into 12 families from more than 1,400 prokaryotic and fungal genomes. Of these, six families have at least one characterized member, whereas other six remain uncharacterized to date. A combination of the PDZ and protease domains is a common scheme of design observed in 88% of the classified proteins. The PDZ–protease domain combination seems to have appeared at a relatively recent time point during the evolution. Prior to this event, the function of the PDZ domain-containing proteins (Fe–S oxidoreductase and Haem-binding uptake family members) was presumably to bind the iron available in large quantities in the early earth atmosphere. The ability of PDZ domains to recognize the unfolded proteins, which are potential substrates for the cellular proteolysis, is likely to have facilitated during their evolution along with the variety of protease domains. The HtrA protease family is encoded by almost all investigated genomes (except few cell wall-less bacteria), whereas it was previously reported in eukaryotes including plants and animals (Ponting and Phillips 1995; Ponting 1997; Sakarya et al. 2010; Clausen et al. 2011; Schuhmann et al. 2012), suggesting that the cellular response to high temperature is universally conserved. The HtrA proteases first diverged from the nonprotease PDZ domain-containing proteins and shared the last common ancestor with remaining protease families. This is consistent with their heat resistance and a requirement to survive the higher temperature of early earth. The DUF3340 domain of Ctp protease family proteins is homologous to many metazoan IRBPs suggesting a link between them. Furthermore, PDZ domains of many Ctp family proteins were predicted to be canonical/metazoan form. This is consistent with their recent divergences evident from the phylogenetic analysis. Of the remaining protein families coined by us, proteins harboring aminopeptidase N, ATP-dependent lon protease, aspartyl protease, and Zn-dependent exopeptidase domains are yet to be characterized functionally, along with the nonproteases Fe–S oxidoreductases and Haem-binding uptake, Tiki superfamily. Crystal structures have been solved for the PDZ domain of aminopeptidase N and ATP-dependent lon protease family member representative IL1258 (UniProt id Q5QTY1) from *Idiomarina loihiensis* and YlbL (UniProt id O34470) from *B.subtilis*, respectively, along with the well-characterized HtrA, Ctp, RseP, and GspC family members*.* The 3D structures of PDZ domains of representative members shown in figure 4 of aspartyl protease, Ctp tricorn protease, Zn-dependent exopeptidase, Haem-binding uptake, Tiki superfamily, sensor histidine kinase ComP, and sporulation protein IV B protease families were modeled to cross-check their authenticity at structural level. Consistent with literature, they exhibit highly conserved fold with a short and a long helix and variable numbers of β-strands (supplementary fig. 10, supplementary file 1, Supplementary Material online). Even with a varying number, β-strands in each structure form a partially opened barrel, and the opening sides of the barrel are each capped with an α-helix as observed in known structures (Doyle et al. 1996). Noticeably, an uncharacterized protein BPSL1254 (UniProt id Q63VJ1) of the aspartyl protease family from *Burkholderia pseudomallei* exhibits best matches to known PDZ domain structures (PDB id 1K32 and 1FC6) of the Ctp protease family members. This clearly supports the recent divergence of aspartyl proteases from Ctp evident in the phylogenetic analysis shown in figure 5*e*. Likewise, the PDZ domain protein DVU_3254 (UniProt id Q726B1), Haem-binding uptake, Tiki superfamily member from *Desulfovibrio vulgaris*, which is placed at the base of HtrA family protein in the phylogenetic tree reveals best match with the HtrA family structures (PDB id 2P3W and 2Z9I). Furthermore, the RMSD values yielded from the structural comparison of predicted and known structures show that the PDZ domain of the AP family has 0.922 RMSD with the canonical metazoan PDZ domain of 1PDR, whereas 2.658 with the eubacterial and 1.701 with the metazoan noncanonical form (supplementary table 2, supplementary file 1, Supplementary Material online). The close match of the AP family structure with canonical metazoan PDZ domain provides additional indirect evidence toward the possible ancestral relationship of Ctp PDZ domains and the canonical metazoan PDZ domains. As stated above, the AP family branches out from Ctp family in our phylogenetic analysis, and a large number of hits to canonical PDZ domain were obtained in Ctp family. On the other hand, the PDZ domain structure of Haem-iron uptake family has 5.030 RMSD value with the canonical form (supplementary table 2, supplementary file 1, Supplementary Material online). It is expected because PDZ domain of Haem-iron update branches out from the Fe–S oxidoreductase family and is likely to be ancestral to the HtrA family members (fig. 5*e*). Taken together, structural comparison strengthens our classification of protein families and identified PDZ domains therein.

In search of their putative functions, we analyzed the genomic neighborhood of all protein-coding genes. Conserved neighborhoods were observed for three families, of which the ATP-dependent lon proteases (structural similarity with tRNA or rRNA modification domain) and Fe–S oxidoreductases are often placed with genes coding for proteins involved in translation and membrane biogenesis, indicating their functions in these processes. RseP intramembrane metalloproteases are already well characterized at experimental levels and are also placed with genes involved in the above-mentioned functions. Such consistent chromosomal proximity of the PDZ domain-coding genes reinforces their roles in translation and membrane biogenesis. If the PDZ domains are indeed involved in translation and membrane biogenesis, the coupling of translation and transcription in prokaryotes might explain the depletion of noncanonical PDZ domains from eukaryotes wherein these processes are spatiotemporally isolated and such domains might not have provided functional advantages. The membranes are the first line of defense for eubacteria against fluctuations in the extracellular milieu. The PDZ domain-containing proteins sense unfolded proteins during various stresses and activate the specialized σ^24^ response. It would be worthwhile to experimentally explore how PDZ proteins modulate translation efficiency and remodel membranes during stress, in addition to their probable role in tRNA and rRNA modifications.

The origin of the PDZ domains was unclear in metazoa as well as in prokaryotes. We hypothesized that the PDZ domain of the Fe–S oxidoreductase family is the probable ancestor for all PDZ domains that might have provided advantage to the ancestral eubacterial species to withstand the anaerobic atmosphere of early earth. Their radical SAM domains might have provided the means of reductive energy generation or translation stability under an anaerobic atmosphere of early Earth. Oxygen availability might have negatively selected these proteins in the species diverged from ancestral gladobacteria but retained them in some extant obligatory anaerobes. On the other hand, proteases might have expanded with the availability of oxygen and helped in adapting to a terrestrial niche. The expansion of PDZ domains in planctomycetes and myxococcus species (debatable host in endosymbiosis theory of first eukaryotic cell formation) is intriguing because both species exhibit many metazoan/eukaryotic like features, that are unusually not found in eubacteria. For instance, planctomycetes have unique internal membrane structures, which could serve as a primitive form of the nuclear membrane and its extension to the endoplasmic reticulum observed in extant eukaryotic cells. On the other hand, myxobacterial species have been used as a model for studying evolution of multicellularity because they form fruiting bodies and have developed mechanisms of coordinated behavior of cells. Furthermore, Ser/Thr/Tyr protein kinases are reported in both species, which are a major class of regulatory and signaling proteins in eukaryotes (Perez et al. 2008). The expansion of the canonical PDZ in metazoans has been correlated with the organismal complexity due to their scarcity/absence in nonmetazoans (Harris and Lim 2001; Sakarya et al. 2010). Incidentally, the number of PDZ domains correlates with complexity/multicellularity even in eubacteria; however, these are noncanonical domains and subset of them might form the fold like canonical, especially the subset of Ctp proteases, which we found to be the youngest family among the others. It has implications not only in the evolution of the eukaryotic cell but also in the evolution of metazoa/multicellularity (Perez et al. 2008; Desmond and Gribaldo 2009; Fuerst and Sagulenko 2011). However, the fungal lineage is a sister clade to metazoan on the phylogenetic tree and lacks a canonical form. Further sequence, structure, and phylogenetic analysis are required to understand the link between eubacterial and fungal PDZ domains with metazoa. Our analysis is limited to completely sequenced prokaryotic and fungal genomes. It is difficult to explain the inheritance and abundance of canonical PDZ domains in metazoan based on data presented here. With high confidence, our analysis confirms the presence of at least three noncanonical PDZ domains in fungi, though they are less likely to possess a gene encoding canonical PDZ. HtrA-like protease family is one of the three which we observed in all fungal genomes. Interestingly, eukaryotic HtrA-like proteases show monophyly with an α-proteobacterial lineage suggesting their mitochondrial origin (Koonin and Aravind 2002). In addition to the HtrA gene, *Saccharomyces cerevisiae* also has a second PDZ domain encoding gene, *NAS2*/YIL007C (UniProt id P40555), which was missed in the earlier genome-scale study in 1997 (Ponting 1997). NAS2 was not classified in one of the families reported here because it has a single PDZ domain similar to HtrA family domain based on Superfamily HMM and to canonical form based on Pfam at the time this analysis was performed. Its PDZ domain adopts noncanonical topology assessed by available crystal structure (PDB id 4O06). NAS2 has been shown to act as a chaperone during the assembly of the 26S proteasome (Funakoshi et al. 2009) and shows sequence and structural similarity with the PDZ domain of the Golgi reassembly-stacking protein (GRASP) encoded by of *GRASP55 and GRASP65* genes, members of the third gene family in fungi which encode PDZ domains. GRASPs contain noncanonical PDZ domain and have been reported in several model eukaryotic organisms except plants (Vinke et al. 2011). In our search also, we identified GRASPs in 16 fungal genomes (supplementary data set 1, supplementary file 2, Supplementary Material online). Further work is needed to understand the evolutionary trajectories of these three fungal PDZ domain-containing proteins in context of protein families investigated in this work and the metazoan counterpart.

In summary, though we provide evidence toward the presence of metazoan-like canonical PDZ domains (in Ctp protease family) in eubacteria, their depletion or absence in archaea and fungi hinders logical explanation for the transition to metazoan phylogeny. Based on the analysis presented here, we argue that archaea presumably never possessed the canonical PDZ domains. A bacterial endosymbiont might have contributed these domains to the eukaryotic phylogeny, wherein they might be lost recurrently only in fungi and ecdysozoa. Acquisition of HtrA family genes in eukaryotic organisms from the α-proteobacterial endosymbiont is a firm example of this possibility. Moreover, phylogenetic analysis confirms the recent divergence of the PDZ domains of the Ctp family, which might have contributed the canonical PDZ form to the metazoan phylogeny. Three PDZ domain-containing genes identified in fungi offer an interesting starting point for further investigation into the relationship between eubacterial, fungal, and metazoan PDZ domains, along with the Ctp family proteases.

## Conclusions

Based on the sequence, structure and phylogenetic analyses presented, we conclude that the PDZ domains of the HtrA family proteins presumably exist across the three domains of life (Archaea, Bacteria, and Eukarya). A large fraction of the PDZ domain-containing proteins remain uncharacterized to date, and this is the first report on their classification into different families. We conclude that the PDZ domains coevolved with the protease domains and are likely to provide functional context to them. Phylogenetic analysis confirms the PDZ domains of the Fe–S oxidoreductases as an ancestral form, whereas the Ctp family domains have diverged recently. Collectively, our comprehensive genomic analysis provides insights into the origin of the PDZ domains and their functional divergence during the evolution.

## Supplementary Material

Supplementary data are available at *Genome Biology and Evolution* online.

## Supplementary Material

Supplementary Data
