# Supplementary material for: PDZ Domains Across the Microbial World: Molecular Link to the Proteases, Stress Response, and Protein Synthesis
Source: Genome Biol Evol. 2019 Jan 29;11(3):644–59. doi: 10.1093/gbe/evz023 (PMC6411480; doi:10.1093/gbe/evz023)
Supplement: Supplementary Data [file evz023_supp.zip › 1.2.Muley_and_Galande_SI.docx]

PDZ Domains across the microbial world: Molecular Link to the Proteases, Stress Response and Protein Synthesis

Vijaykumar Yogesh Muley^1,2,*^, Yusuf Akhter^3^ and Sanjeev Galande^2,*^

^1^ Instituto de Neurobiología, Universidad Nacional Autónoma de México, Querétaro, Mexico

^2^ Department of Biology, Indian Institute of Science Education and Research, Pune, India

^3^ Department of Biotechnology, Babasaheb Bhimrao Ambedkar University, Lucknow, India

* To whom correspondence should be addressed.

Email: [vijaykumar.muley@outlook.de](mailto:vijaykumar.muley@ucc.ie), [vijay.muley@comunidad.unam.mx](mailto:vijay.muley@comunidad.unam.mx) (VYM); [sanjeev@iiserpune.ac.in](mailto:sanjeev@iiserpune.ac.in) (SG)

Supplementary Information

Supplementary Tables

**Supplementary Table 1.** **The distribution of canonical PDZ (Metazoan PDZ-like) domain variants in various phyla/classes.** Canonical PDZ domains are observed in Ctp and Htr family proteins only. The domains are present in notable numbers in Ctp family across several bacterial groups. For high confidence, a predicted PDZ domain was considered canonical variant if it was predicted by both Superfamily and Pfam Hidden Markov Model of canonical PDZ variant.

| Kingdom | Phylum | # of Ctp proteins/  Species | # of Htr proteins/  Species |
| --- | --- | --- | --- |
| Eubacteria |  | 362/330* | 18/17 |
|  | Alphaproteobacteria | 13/13 | 5/5 |
|  | Bacteroidetes/Chlorobi | 53/43 | - |
|  | Chlamydiae/Verrucomicrobia | 19/16 | - |
|  | Cyanobacteria | 14/9 | - |
|  | Deltaproteobacteria | 11/9 | 1/1 |
|  | Gammaproteobacteria | 246/235 | - |
|  | Planctomycetes | 6/5 | 2/1 |
| Fungi |  | - | 10/10 |
|  | Ascomycota | - | 10/10 |

^*Metazoan PDZ domain-like sequence along with DUF3340 domain was found in 309 Ctp proteins in 298 eubacterial species.^

**Supplementary Table 2. Comparison of modelled and known structures of non-canonical and canonical PDZ domains**

| NCBI  Locus ID | UniProt accession | Source organism | Modelled  Region | Predicted family in this work | 2Z9I | 1LCY | 1PDR |
| --- | --- | --- | --- | --- | --- | --- | --- |
| BPSL1254 | Q63VJ1_BURPS | *B. pseudomallei* | 284-357 | AP | 2.658 | 1.701 | 0.922 |
| DVU3254 | Q726B1_DESVH | *D. vulgaris* | 368-447 | Haem-iron uptake | 0.584 | 0.798 | 5.030 |
| RB6120 | Q7UQS9_RHOBA | *R. baltica* | 987-1070 | ZEP | 0.639 | 0.468 | 2.122 |
| BSU24230 | SP4B_BACSU | *B. subtilis* | 123-211 | SpoIVB | 6.628 | 0.575 | 1.899 |
| SRU_2235 | Q2S0E1_SALRD | *S. ruber* | 795-867 | Ctp | 0.722 | 0.589 | 1.049 |
| BSU31690 | COMP_BACSU | *B. subtilis* | 31-103 | ComP | 1.147 | 3.394 | 0.747 |

^Notes: 2Z9I PDB structure represents non-canonical structure from^ *^Mycobacterium tuberculosis^* ^belonging to HtrA family, 1LCY is a metazoan counterpart of the HtrA family from human, whereas 1PDR represents the canonical third PDZ domain of the human homolog of Discs Large Protein. Structure of AP family representative was modelled using full length protein sequence. Abbreviations used are^ **^AP^**^-Aspartyl protease,^ **^ZEP^**^-Zinc-dependent exopeptidase,^ **^SpoIVB^**^-Sporulation protein IV B,^ **^Ctp^**^-C-terminal processing protease,^ **^ComP^**^-Sensor histidine kinase competence protein^

Supplementary Figures

| 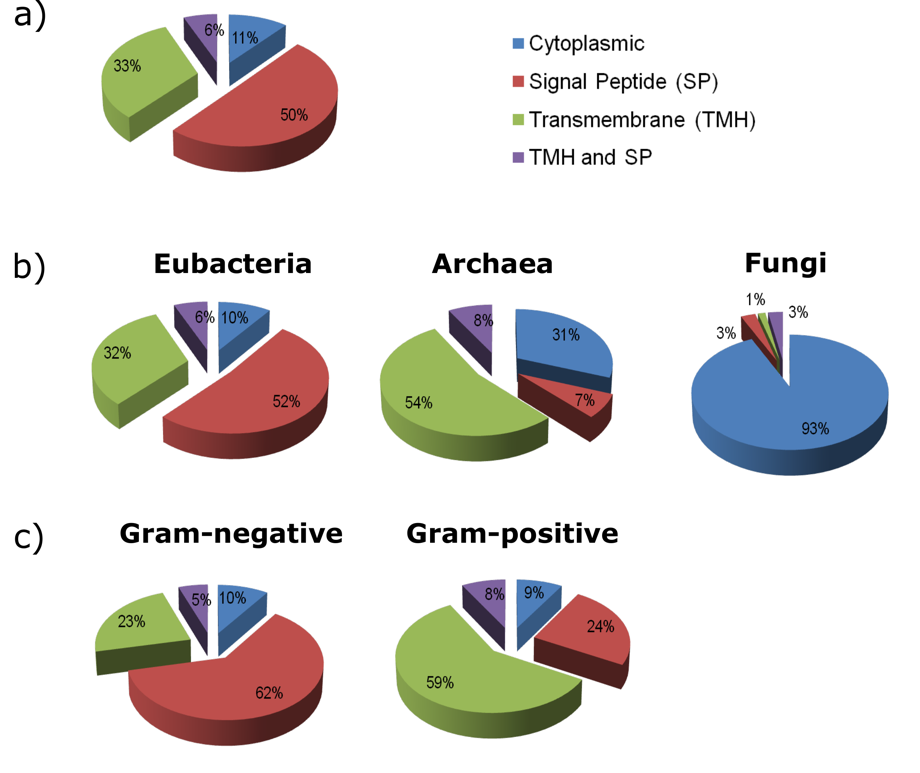 |
| --- |
| **Supplementary Figure 1. Sub-cellular localization of PDZ domain-containing proteins.** The pie chart shows percentages of the PDZ-containing proteins as per their predicted sub-cellular localization for **(a)** all **(b)** eubacterial, archaeal and fungal, **(c)** Gram-negative and –positive proteins. Large fractions of proteins are predicted to be targeted to membrane compartments and only 11% are cytoplasmic. Eubacterial proteins are predicted primarily to harbor signal peptides; archaeal proteins are mainly localized in membranes and fungal proteins in cytoplasm. Gram-negative proteins are mainly predicted to have signal peptides whereas Gram-positive proteins are predominantly localized in membranes. |


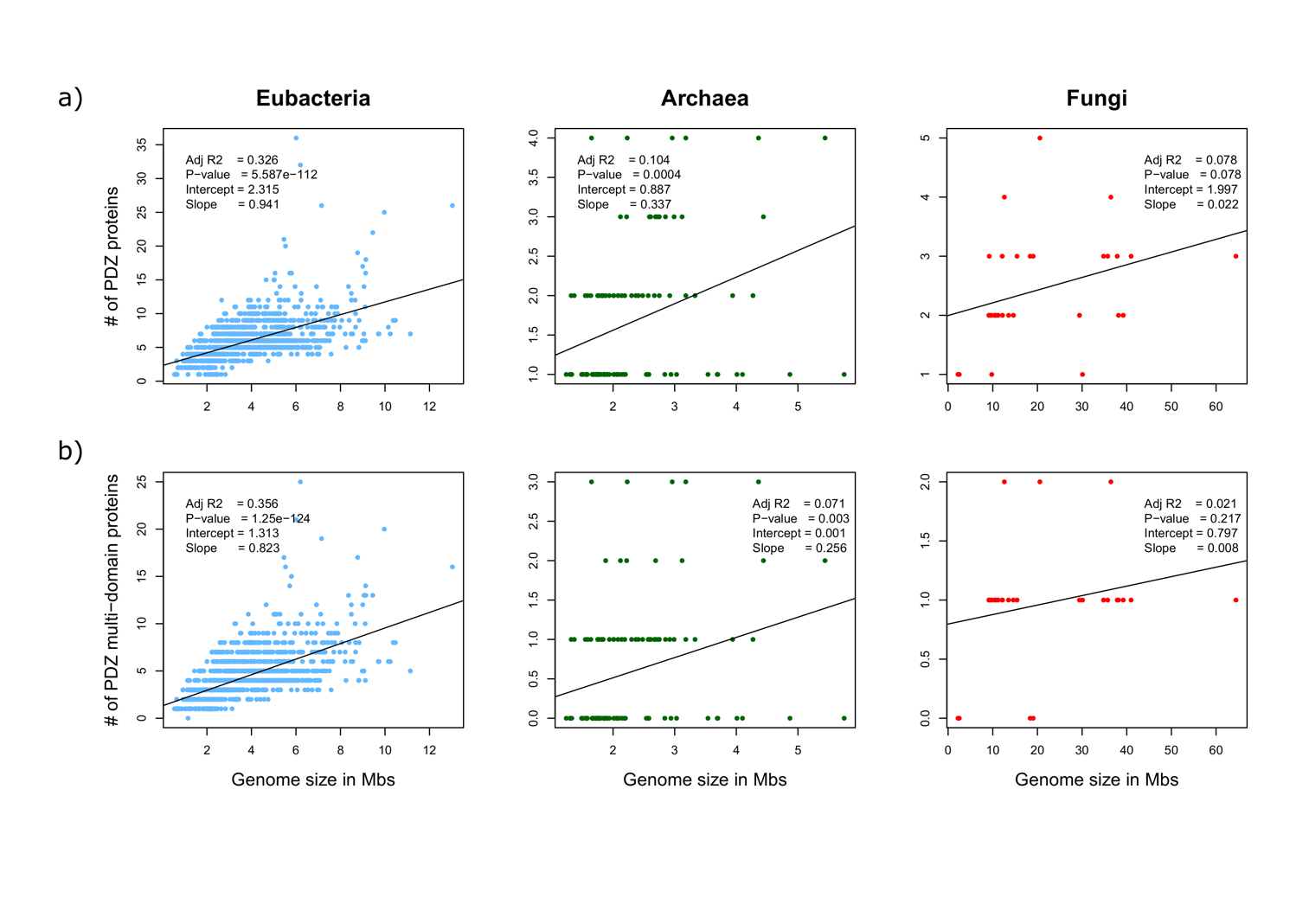


| **Supplementary Figure 2. Expansion of PDZ domain-containing proteins correlates with genome size in eubacteria.** Relationship of genome size with a number of PDZ domain-containing proteins **(a)** and with a number of multi-domain proteins **(b)** in eubacteria, archaea and fungi. Linear regression is indicated with solid line. For eubacteria, in addition of statistical significant p-value for the linear regression, a positive correlation between the genome size and both number of PDZ domains and number of PDZ multi-domain proteins can be observed. The correlation is notably weaker in archaea, still the linear regression is significant for genome size relation to both number of PDZ proteins and number of PDZ multi-domain proteins. In contrast, either of these correspondences shows a near-random correlation in fungi, and linear regression here is also not significant. |
| --- |

| 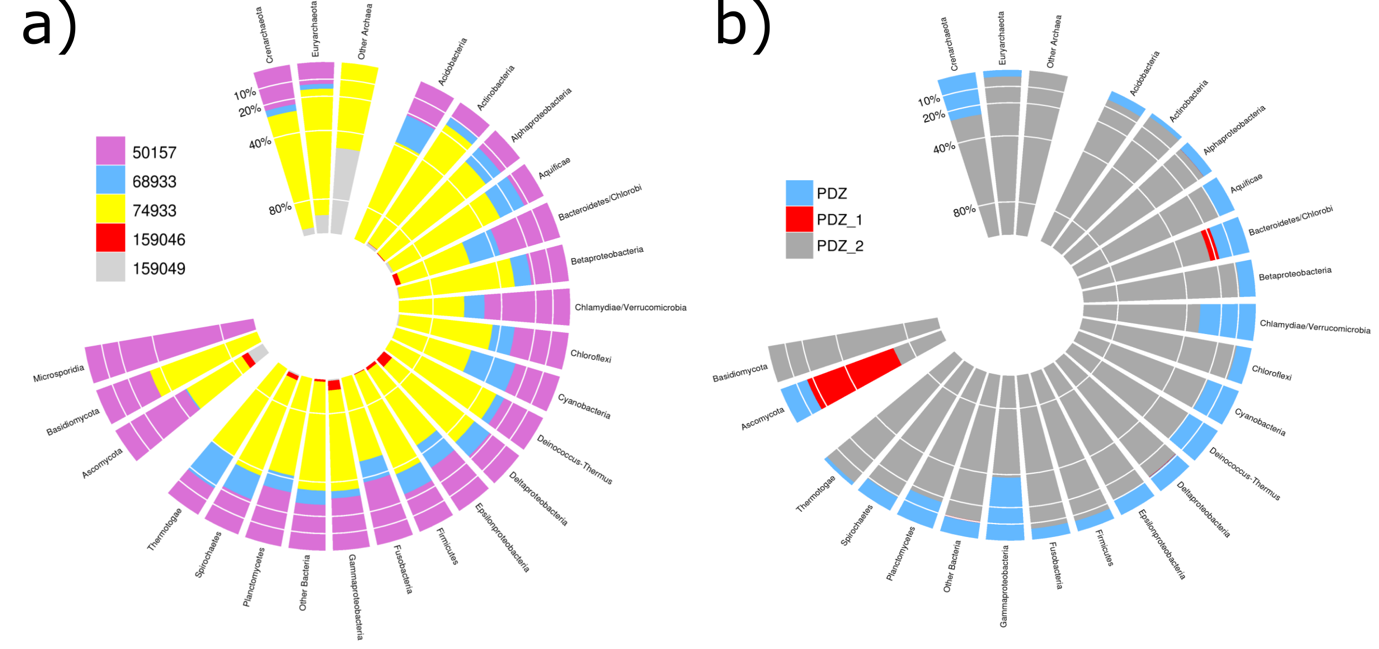 |
| --- |
| **Supplementary Figure 3. Proportion of PDZ variants in various phyla/classes of archaea, eubacteria and fungi.** Estimates on percentages of each PDZ variant with respect to their overall distribution in specific groups of genomes is shown in the form of polar histograms. Domains predicted with Superfamily HMM model and its sub-family level distribution is shown in (a) and predicted with Pfam HMM model in (b). The HMM nomenclature is according to Pfam and Superfamily database accession identifiers. Superfamily accession stands for 50157 : PDZ domain, 68933 : Tail specific protease PDZ domain, 74933 : HtrA-like serine proteases, 159046 : EpsC C-terminal domain-like, 159049 : MTH1368 C-terminal domain-like. The canonical domain form (which is expanded in metazoa), denoted as 50157 in (a) and PDZ in (b), is the second most abundant domain in eubacteria, ascomycota (fungi) and crenarchaeota (archaea). The non-canonical domain form (which is found in eubacteria and plants) denoted as 74933 in (a), as PDZ_2 in (b), is expanded in eubacteria. |


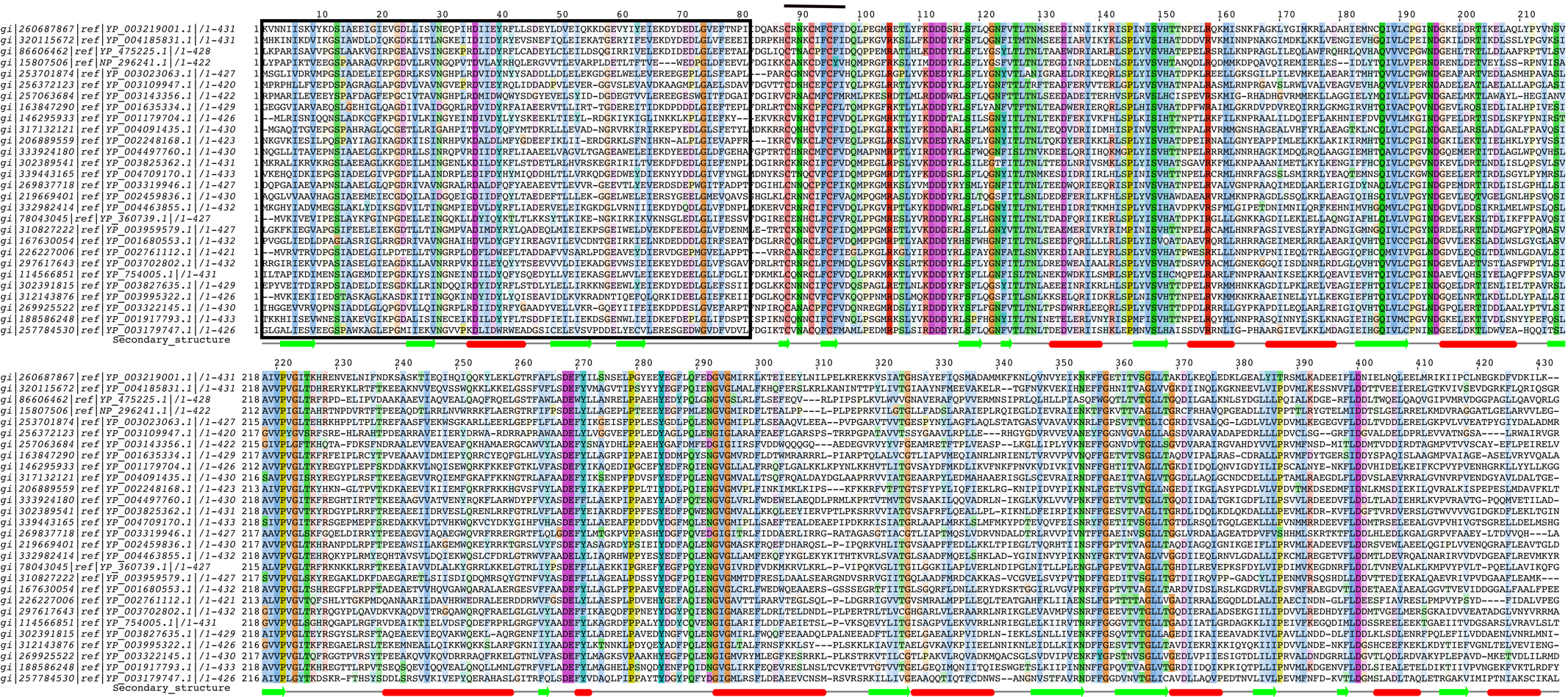


**Supplementary Figure 4. Multiple sequence alignment of Fe-S oxidoreductase family proteins.** The figure shows a multiple sequence alignment (MSA) of 28 representative sequences selected from the 155 proteins of Fe-S oxidoreductase superfamily at 50% sequence similarity level. MSA is consists of 433 sites after removal of positions with gaps in more than 30% of total sequences. The alignment shows a PDZ domain (shown withing rectangular border) which can be recognized by Superfamily HMM only. The CxxxCxxC iron-sulfur binding motif is highly conserved in this superfamily and highlighted with black line on the top. Secondary structural elements are shown in cartoon at the bottom of MSA. Red color rods represent helices whereas beta-sheets are depicted in green color arrowheads.

| 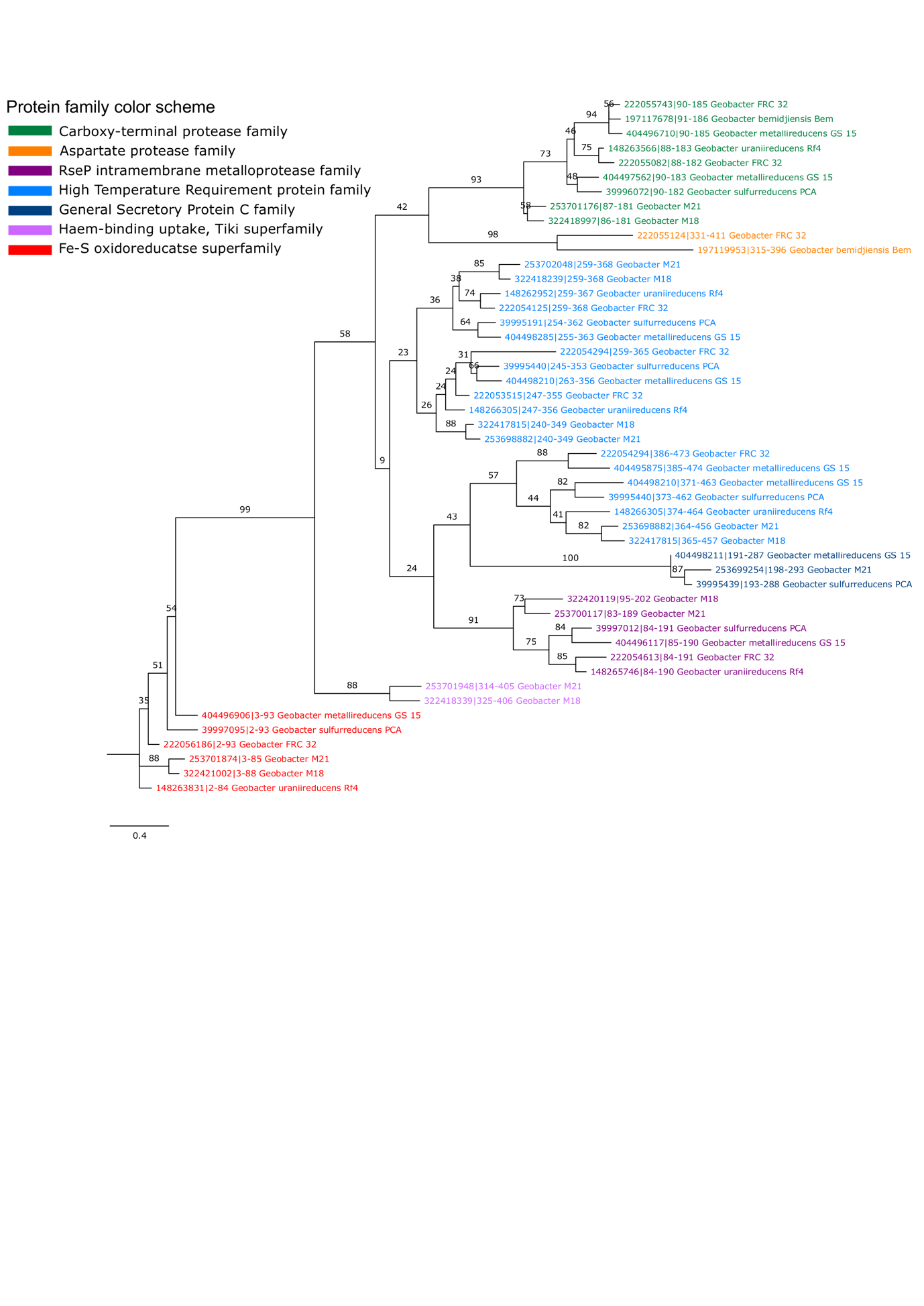 |
| --- |
| **Supplementary Figure 5. Maximum likelihood phylogenetic tree of PDZ domain variants from various families proposed in our study.** Phylogenetic tree was constructed using RAxML v. 8.1.24 [1], as implemented on the CIPRES web server [2], under the WAG plus gamma model of evolution, and with the number of bootstraps automatically determined (MRE-based bootstopping criterion). A total of 660 bootstrap replicates were conducted under the rapid bootstrapping algorithm, with 100 sampled to generate proportional support values. The tree was visualized in FigTree1.4 (available from <http://tree.bio.ed.ac.uk/software/figtree/>) with levels of support shown as bootstrap values. Phylogenetic tree supports the ancestry of PDZ domains of Fe-S oxidoreductase family whereas recent divergence of Ctp family domains. |

| 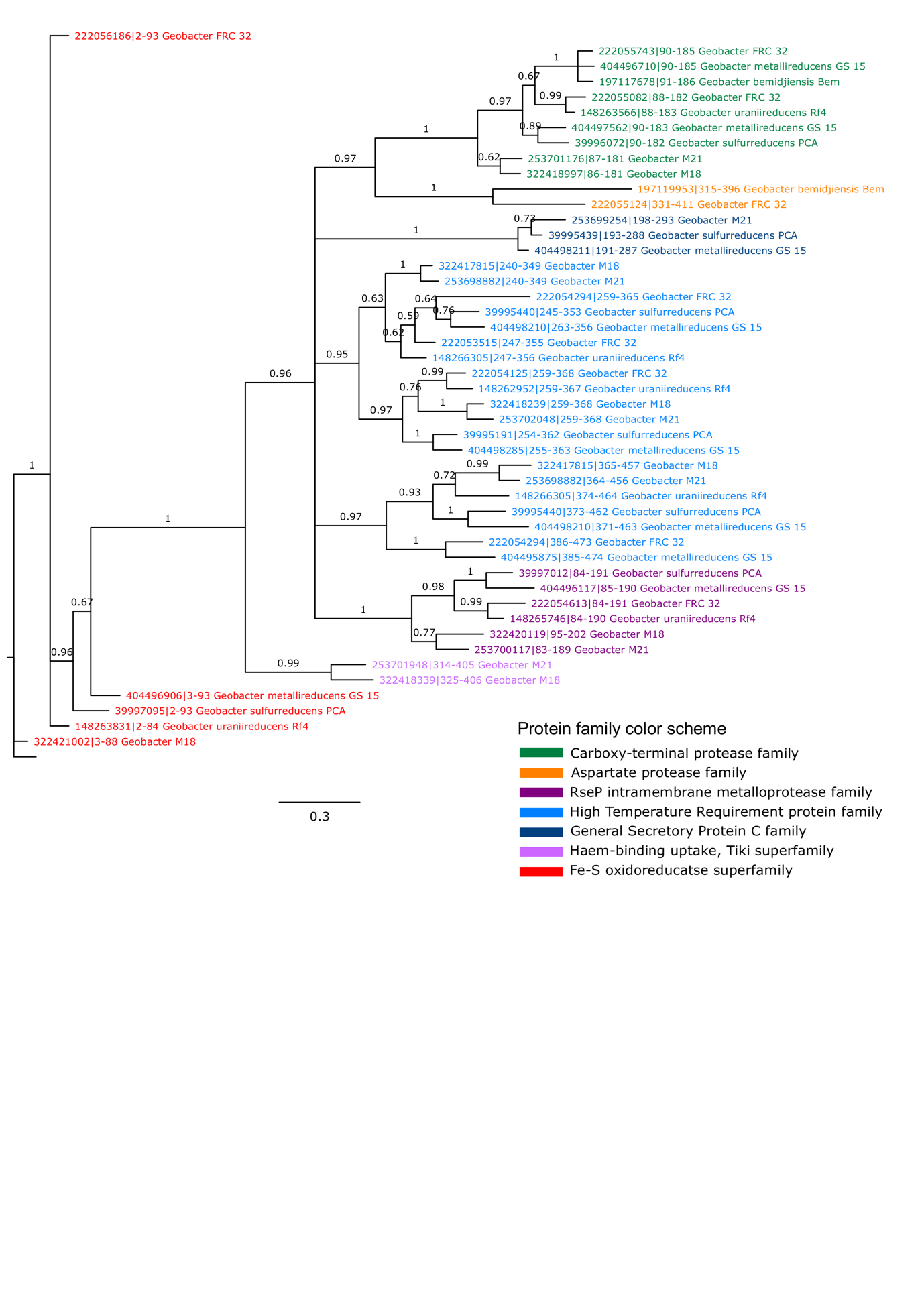 |
| --- |
| **Supplementary Figure 6. Bayesian phylogenetic tree of PDZ domain variants from various families proposed in our study.** Phylogenetic tree was reconstructed using MrBayes [3]. Bayesian analysis was performed for one million generations with WAG substitution model and gamma distribution for four categories. The trees were sampled after every 1000 generations and the first 25% was discarded as burn-in. The tree was visualized in FigTree1.4 (available from <http://tree.bio.ed.ac.uk/software/figtree/>) with levels of support shown as posterior probabilities. Phylogenetic tree supports the ancestry of PDZ domains of Fe-S oxidoreductase family whereas recent divergence of Ctp family domains. |

| 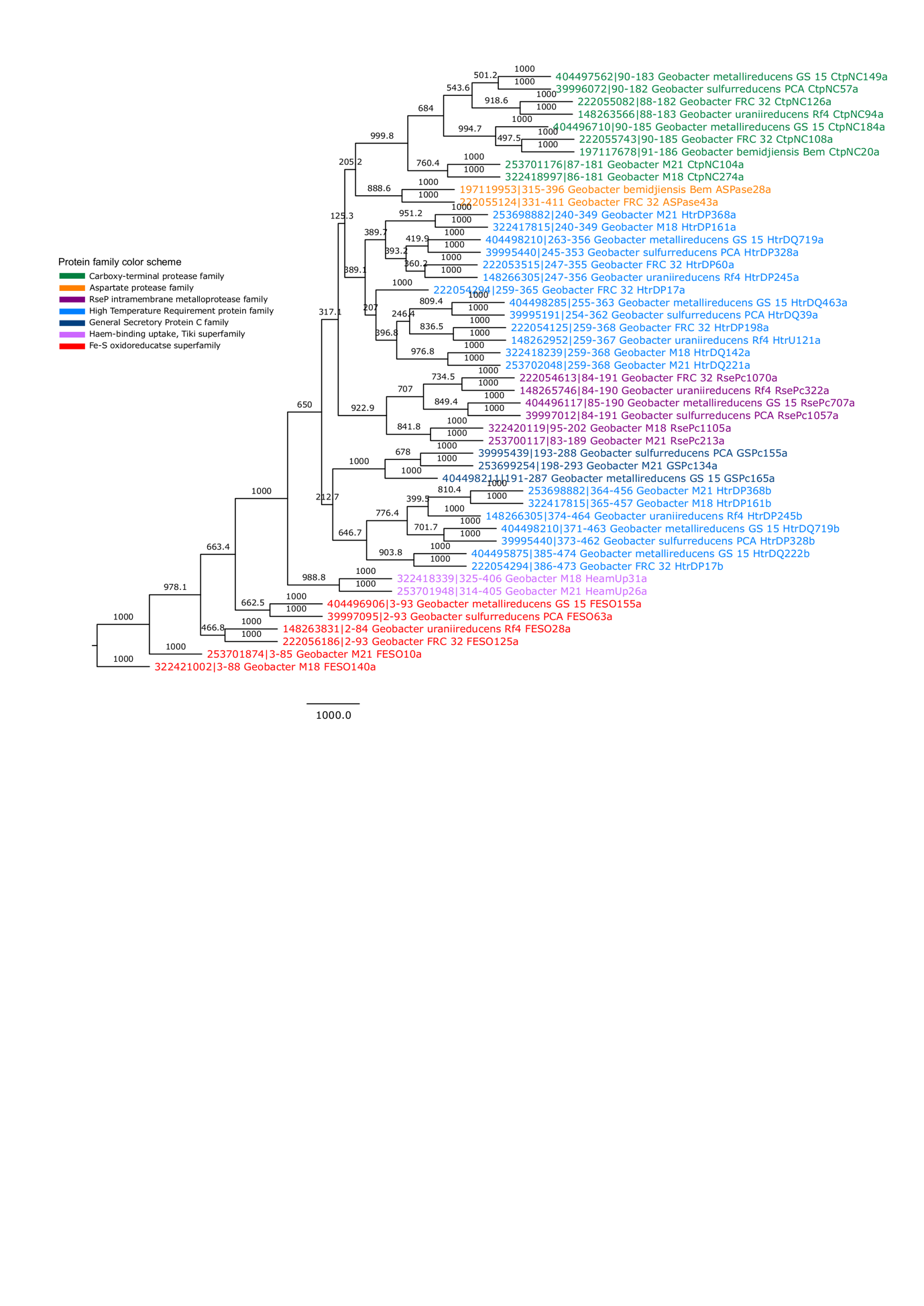 |
| --- |
| **Supplementary Figure 7. Maximum parsimony phylogenetic tree of PDZ domain variants from various families proposed in our study.** Phylogenetic tree was reconstructed with parsimony algorithm available through *protpars* program in Phylip package [4]. The statistical significance was accessed with 1,000 bootstraps. The tree was visualized in FigTree1.4 (available from <http://tree.bio.ed.ac.uk/software/figtree/>) with levels of support shown as bootstrap values. Phylogenetic tree supports the ancestry of PDZ domains of Fe-S oxidoreductase family whereas recent divergence of Ctp family domains. |

.

**
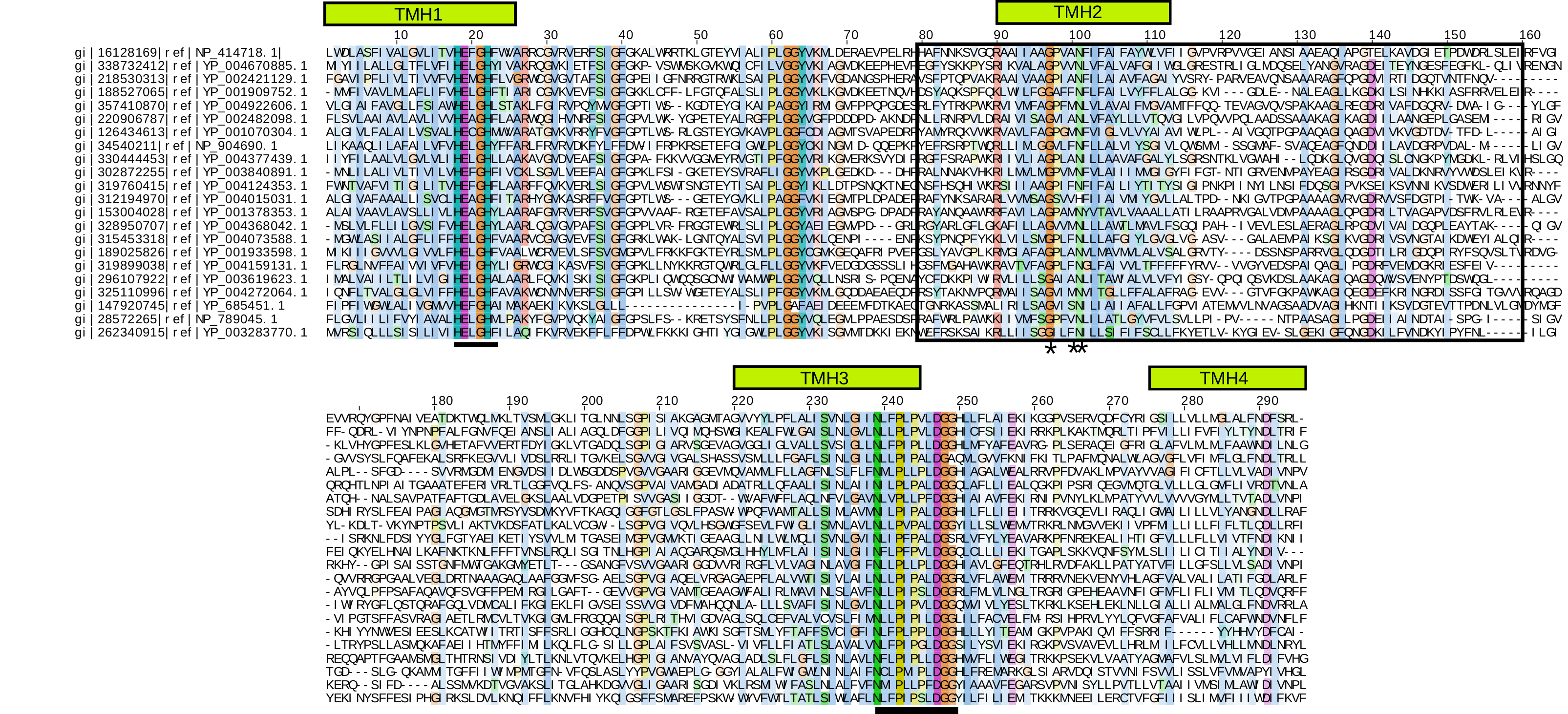
**

**Supplementary Figure 8. Multiple sequence alignment of Regulator of Sigma-E Protease (RseP) family proteins.** The figure shows a multiple sequence alignment (MSA) of 22 representative sequences selected from the 1,373 proteins of RseP family at 50% similarity level. MSA consists of 296 sites after removal of positions with gaps in more than 30% of total sequences. The alignment shows a PDZ domain (shown within rectangular border) and four predicted transmembrane helices (TMH). The prototype motifs conserved in all family members are highlighted with black line at the bottom. The residues required for PDZ domain activity are shown with asterisks. Color intensities of amino acids represent conservation level. TMH are conserved among other sites and required for protein function.


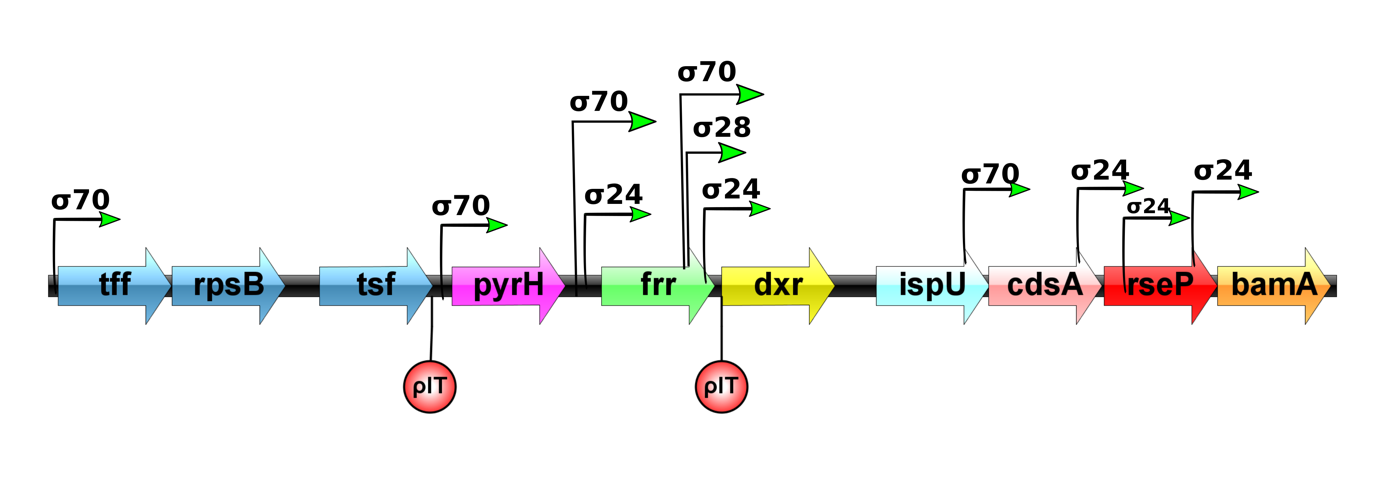


**Supplementary Figure 9. A schema of genomic context of Regulator of Sigma-E Protease (RseP) coding gene in *Escherichia coli*.** Genes belonging to the same transcriptional units are depicted in same colors. Promoter sites of sigma factors are shown with flagged green arrows. Rho-independent termination sites (ρIT) are shown with red bubbles. Genes and intergenic regions are not on scale. All these genes are placed within an intergenic distance of 300 nucleotide bases between them. Based on promoter and termination sites, this genomic region is expected to form at least three transcription units: First, the *tff-rpsB-tsf* genes and second the *pyrH-frr* genes encoding products involved in protein synthesis which are likely to be regulated by σ^70^; and the σ^24^ regulated *dxr-ispU-cdsA-rseP-bamA* genes coding for membrane components. EcoCyc database was used to collect regulatory information.


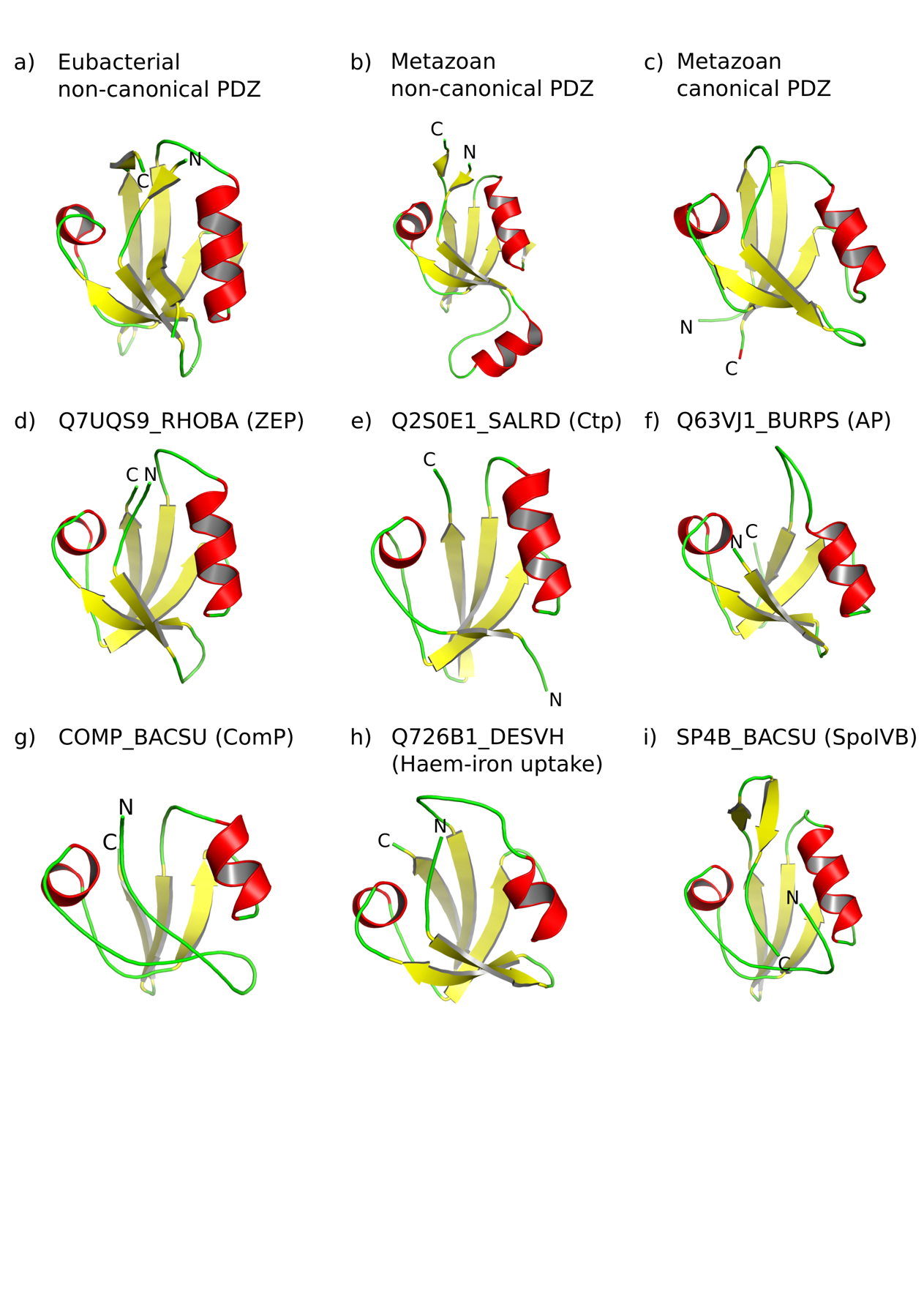


**Supplementary Figure 10. Structural comparison of known and predicted PDZ domain structures. a)** Crystal structure of non-canonical PDZ domain of RV0983 protein (HtrA) from *Mycobacterium tuberculosis*, PDB id is 2Z9I **b)** Non-canonical PDZ domain structure of the mitochondrial HtrA2 from human, PDB id is 1LCY **c)** Canonical PDZ domain structure of the third PDZ domain of the human homolog of Discs Large Protein, PDB id is 1PDR **d-i)** Predicted structures of representative PDZ domains from uncharacterized protein annotated with their UniProt identifiers and identified families in parenthesis. Abbreviations used are **ZEP**-Zinc-dependent exopeptidase, **Ctp**- C-terminal processing protease with tricorn domain, **AP**-Aspartyl protease, **ComP**-Sensor histidine kinase competence protein, **SpoIVB**-Sporulation protein IV B. All predicted structures have a highly conserved fold similar to available structures, with two helices and varying numbers of beta-strands. Details of proteins used for structure prediction (d-i) is given in the Supplementary table 2 along with RMSD values obtained from alignment with known structures shown in (a-c).

References

1. Stamatakis, A., *RAxML-VI-HPC: maximum likelihood-based phylogenetic analyses with thousands of taxa and mixed models.* Bioinformatics, 2006. **22**(21): p. 2688-90.

2. Miller, M.A., Pfeiffer, W., and Schwartz, T., *Creating the CIPRES Science Gateway for inference of large phylogenetic trees.* Proceedings of the Gateway Computing Environments Workshop (GCE), 2010: p. 1-8.

3. Huelsenbeck, J.P. and F. Ronquist, *MRBAYES: Bayesian inference of phylogenetic trees.* Bioinformatics, 2001. **17**(8): p. 754-5.

4. Felsenstein, J., *PHYLIP - Phylogeny Inference Package (Version 3.2).* Cladistics, 1989. **5**: p. 164-166.
