# Supplementary material for: PDZ Domains Across the Microbial World: Molecular Link to the Proteases, Stress Response, and Protein Synthesis
Source: Genome Biol Evol. 2019 Jan 29;11(3):644–59. doi: 10.1093/gbe/evz023 (PMC6411480; doi:10.1093/gbe/evz023)
Supplement: Supplementary Data [file evz023_supp.zip › Supplementary legends.docx]

Supplementary files

Filename: Additional File 1
Title of data: Supplementary information
Description of data: Supplementary figures and tables

Filename: Additional File 2
Title of data: Supplementary dataset 1
Description of data: NCBI identifiers, classification, domain coordinates, and architecture of the PDZ domain-containing proteins identified in this study.

Filename: Additional File 3
Title of data: Supplementary dataset 2
Description of data: NCBI identifiers, taxonomy, genome size and phenotype information of 1,474 species used in this study.
